# Integrating single-cell RNA sequencing and spatial multi-omics reveals the molecular signature of regeneration after spinal cord injury

**DOI:** 10.1186/s40364-025-00845-4

**Published:** 2025-10-21

**Authors:** Haoru Dong, Yuanqing Ding, Xingyu Chen, Xiao Xiao, Longnian Zhou, Haiyue Lin, Zezhen Zhang, Yiming Tao, Shiyi Cai, Jianlan Zhao, Xiaomu Li, Rong Xie

**Affiliations:** 1 Department of Neurosurgery, National Center for Neurological Disorders, Shanghai Clinical Medical Center of Neurosurgery, Shanghai Key Laboratory of Brain Function and Restoration and Neural Regeneration, Huashan Hospital, Neurosurgical Institute of Fudan University, Fudan University, Shanghai, 200040 China; 2 Department of Endocrinology, Zhongshan Hospital, Fudan University, Shanghai, 200032 China; 3 Department of Neurosurgery, National Regional Medical Center, Huashan Hospital Fujian Campus, Fudan University, The First Affiliated Hospital Binhai Campus of Fujian Medical University, Fuzhou, Fujian 350209 China

**Keywords:** Single-cell RNA sequencing, Spatial transcriptomics, Spatial metabolomics, Spinal cord injury, Regeneration

## Abstract

**Background:**

A certain degree of self-repair is initiated following spinal cord injury (SCI). Although intraneuronal regeneration and a supportive growth environment are limited, they serve as the foundation for functional recovery after SCI.

**Methods:**

In this study, we conducted single-cell RNA sequencing combined with spatial transcriptomics and spatial metabolomics to reveal the spatial molecular characteristics of self-repair processes after SCI at single-cell resolution.

**Results:**

We identified three cell subsets-Mic2 (a microglia subset), Mac4 (a macrophage subset), and Fib4 (a fibroblast subset)-that express markers associated with spinal cord repair. Mic2 and Mac4 exhibit clustered spatial distribution patterns, whereas Fib4 is predominantly located around the injured spinal cord. Additionally, Mic2 is predominantly distributed in the white matter, particularly in the dorsal region of the injured spinal cord, and exhibits high expression of taurine. Mac4 and Fib4 exhibit high expression of copalic acid and uridine, respectively.

**Conclusions:**

In this study, we have identified three distinct cell subsets that express markers associated with wound healing and may promote regenerative processes, and we have determined their spatial transcriptional and metabolic features enriched within these regions. Our dataset represents a valuable resource that offers novel mechanistic insights into the pathobiology of spinal cord injury.

**Supplementary Information:**

The online version contains supplementary material available at 10.1186/s40364-025-00845-4.

## Background

Spinal cord injury (SCI) is a devastating condition that can result in permanent disability and loss of function [1, 2]. Hemorrhage, edema, necrosis of the neural tissue occur following SCI. Subsequently, a cascade of molecular and cellular events exacerbates the initial damage and contributes to additional tissue loss [3, 4]. Following the acute phase of SCI, the damaged spinal tissue initiates a self-repair process [5]. For instance, the expression levels of nerve growth factors increase, indicating the presence of self-repair mechanisms after SCI [6]. However, our current understanding of intraneuronal regeneration and the supportive growth environment remains limited, particularly at single-cell and spatial resolution [7, 8]. 

Single-cell RNA sequencing (scRNA-seq) is an innovative and rapidly advancing high-throughput sequencing technology that enables a comprehensive exploration of gene expression and functional characteristics at the single-cell level [9]. It can help distinguish rare subsets and identify unknown cell types, thereby providing novel insights and tools for understanding the complexity of biological systems [10]. Numerous researchers have investigated changes in cellular composition and molecular functions following SCI [11, 12]. For instance, Milich et al. studied the cellular heterogeneity and interactions in the injured spinal cord at 1, 3, and 7 days post-injury and identified the molecular signatures of angiogenesis, gliosis, and fibrosis after SCI [11]. However, current single-cell studies of SCI lack structural and spatial information on gene expression and its functional outcomes (e.g., metabolic changes). The emergence of spatial omics addresses the limitations of scRNA-seq. For example, Zheng et al. studied the transcriptomic regulation and metabolic changes in the damaged brain using spatial multi-omics and found that neuronal loss was spatially correlated with lipid and myo-inositol metabolism [13]. In their study, spatial omics clarified the localization of injured neuronal cells and uncovered the significance of lipid and myo-inositol metabolism. However, in-depth investigations of metabolic changes in SCI at the spatial and single-cell levels remain unclear.

Therefore, scRNA-seq, spatial transcriptomics (ST), and spatial metabolomics (SM) were carried out on a rat SCI model. The data revealed that the major cell types involved in spinal cord repair included microglia, macrophages, and fibroblasts. Spatial analysis revealed that regeneration-associated microglia, macrophages, and fibroblasts aggregate into clusters, and these areas accumulate pro-regenerative metabolites. The findings of this study provide a multi-omics framework for identifying therapeutic targets for SCI and contribute to an improved understanding of the heterogeneity and microenvironment of SCI.

## Methods

### Animal model

All experimental procedures were conducted in accordance with the Guide for the Care and Use of Laboratory Animals Guide and approved by Animal Ethics Committee of Fudan University Shanghai Medical College. Eight-week-old male Sprague-Dawley rats, weighing between 200 and 250 g, were used. Eight-week-old male mice were used for validation. The compression SCI model was used as previously described [14]. Briefly, animal operation was performed under isoflurane anesthesia (2%) and a 1:1 mixture of O2 (RWD Life Science Co. R510–22–16, China). After disinfecting, the rat underwent T1 laminectomy and then performed compression injury by quick-release a 50 g clip for 10s. For the sham group, only laminectomy was performed without SCI. Animals were monitored daily for infections and abnormal weight loss. For the copalic acid (CA; BioBioPha, BBP06444, China) treatment group, mice were administered a dose of 1 mg/kg body weight via local injection. For the recombinant Igf2 (rIgf2, CUSABIO, CSB-YP011088MO, China) treatment group, mice received an local injection of rIgf2 (100 ng/µl; 2 µl) immediately after injury. The control group was injected with an equal amount of PBS. The bladder was manually expressed twice daily to assist with urination.

### Behavioral assessment

Post-injury hindlimb functional recovery was evaluated weekly using three distinct methods: the Basso Mouse Scale (BMS) for locomotor scoring, the inclined plane test for assessing balance and strength, and the CatWalk XT automated gait analysis system for detailed motion tracking. During open-field testing, experimental mice were allowed to move freely for four minutes, with their hindlimb movements scored on a scale from 0 (no observable movement) to 9 (normal locomotion). To ensure unbiased assessment, independent observers, blinded to experimental groups, performed the scoring. The inclined plane test measured neuromuscular function by placing mice on an adjustable ramp. The maximum angle at which each animal could maintain stability for 10 s without slipping was recorded as an indicator of limb strength and coordination. For high-resolution gait analysis, the CatWalk XT system was employed. This automated platform captures dynamic paw placement, stride length, step sequence, and pressure distribution across the hindlimbs while mice traverse a glass walkway. Infrared lighting and high-speed cameras recorded footprints, allowing for quantitative assessment of parameters such as stance duration, swing speed, and interlimb coordination. This system provides objective, computerized metrics to detect subtle changes in locomotion that may not be evident in manual scoring. Each experimental group consisted of at least six animals to ensure statistical reliability.

### Tissue processing

All rats were euthanized by an overdose of isoflurane anesthesia. For sequencing samples, physiological saline was used for irrigation and was collected a 1 cm spinal cord segment centered on the lesion core. For immunohistochemistry samples, physiological saline and 4% polyformaldehyde were used for irrigation. Then the spinal cord was collected and placed in 4% polyformaldehyde overnight at 4 °C, following by transferred to 30% sucrose for dehydration, encapsulation in OCT on dry ice, and stored at −80℃. For single-cell sequencing and spatial sequencing samples, only physiological saline was used for irrigation. The tissues were placed in single-cell preservation solution or encapsulated in OCT. Due to the limited availability of spinal cord injury samples, only the locally injured tissue provided sufficient material for scRNA-seq. As a result, scRNA-seq and spatial multi-omics sequencing (ST and SM) were performed on separate samples. Furthermore, to match the results of ST and SM, adjacent slices were used for ST and SM analyses separately.

### Cell culture and LPS-induced inflammatory model

The BV2 and RAW264.7 cell lines were obtained from the Cell Bank of the Chinese Academy of Sciences. These cells were cultured in DMEM with 10% FBS, 100 µg/ml streptomycin, and 100U/ml penicillin. They were maintained in a humid atmosphere at 37℃ with a mixture of 95% air and 5% CO2. LPS (1 µg/ml) and copalic acid (CA; BioBioPha, BBP06444, China) were added to BV2 and RAW264.7 cultures for 6 h. For the CA treatment group, a concentration of 3 µM was used for cell culture referring to the result of SM.

### qRT-PCR

The total RNA from cells was isolated using Trizol reagent according to the instructions provided by the manufacturer (Invitrogen, USA). Reverse transcription was conducted using the ReverTra Ace qPCR RT Master Mix (Toyobo, FSQ-201, China). The RNA concentrations were adjusted to 1.2 mg/mL in nuclease-free water, and then the total RNA was reverse-transcribed in a 20 µL reaction volume. The cDNA was amplified by real-time quantitative RT-PCR using the SYBR Green reagent (Roche, Switzerland). The samples were assayed in triplicate, and β-Actin was utilized as the internal control. The primer sequences used for qPCR in this study are as follows: β-Actin primer: forward 5’-GGCTGTATTCCCCTCCATCG-3’ and reverse 5’-CCAGTTGGTAACAATGCCATGT-3’. CD86 primer: forward 5’-CTGGACTCTACGACTTCACAATG-3’ and reverse 5’-AGTTGGCGATCACTGACAGTT-3’. CD80 primer: forward 5’-ACCCCCAACATAACTGAGTCT-3’ and reverse 5’-TTCCAACCAAGAGAAGCGAGG-3’. IL-1β primer: forward 5’-GCAACTGTTCCTGAACTCAACT-3’ and reverse 5’-ATCTTTTGGGGTCCGTCAACT-3’.

### Immunohistochemistry

Spinal cord tissues were sectioned into 20-µm-thick sections. These sections underwent triple washes with PBS and were subsequently fixed using freshly prepared 4% PFA for a duration of 30 min. Antigen retrieval was facilitated by exposing the sections to sodium citrate antigen retrieval solution (Solarbio) for 30 min at 90 °C. A solution containing 0.3% Triton X-100 and 5% BSA was utilized for blocking, carried out at room temperature. Subsequently, the sections were subjected to overnight incubation with primary antibodies at 4 °C. The primary antibodies employed were anti-ARG1 (CST, #93668), anti-iNOS (proteintech, CL647-18985), anti-NeuN (Abcam, ab104224), anti-GFAP (Santa Cruz, sc-33673), anti-5HT (Immunostar, 20080), anti-Igf2 (Absin, abs115963), and anti-Col1a1 (Santa, sc-293182). After a triple wash with PBS, the sections underwent a 1-hour incubation with the respective secondary antibodies (1:1,000, Invitrogen). The obtained images were captured using a SLIDEVIEW VS200 microscope (Olympus, Germany), as well as confocal microscopes (Nikon, Japan). Image analysis was conducted using either Image J or Adobe Photoshop CC 14.0 software.

### Single-cell sequencing

For comprehensive details regarding the scRNA-seq procedure for spinal cord tissues, the specific protocol can be accessed on the 10X website. Fresh samples of spinal cord tissue were gathered, and within a span of 12 hours, single-cell suspensions were extracted using the stipulated assay. Following this, construction of the single-cell 3’ library was executed in strict accordance with the 10X Chromium protocol (10X Genomics Chromium Single Cell 3′ Reagent Kit v3, PN-1000121). Subsequently, each individual single-cell 3’ library underwent sequencing utilizing the Illumina Novaseq 6000 platform.

Data processing and visualizations were performed using the Seurat (version 4.3.0) package [15]. For each sample, genes expressed in fewer than three cells and cells with fewer than 200 genes were removed. The package DoubletFinder was used to remove doublets [16]. Cells with a number of features larger than 500 and less than 7,000, RNA counts less than 60,000, mitochondrial expression level less than 15% were used. The LogNormalize method, with a scale factor of 10,000, was utilized for normalization. The FindVariableFeatures function was then employed to extract the top 3,000 variable features. Data were subsequently scaled based on mitochondrial percentage, using the ScaleData function. Clustering results were visualized using UMAP plots. The function FindAllMarkers was employed to identify significant genes within each cluster. Cell types were determined by employing marker genes sourced from relevant literature. For functional enrichment analysis, the clusterProfile package was utilized [17]. Pseudotime analysis was performed using Monocle2 package [18]. CellChat package was used to construct the cell-cell communication network [19]. Unless otherwise indicated, default parameters were used for all analyses. For Li’s and Milich’s scRNA-seq datasets, the parameters of the published articles were used.

### Spatial transcriptomics

The frozen OCT blocks were sectione at a thickness of 10 μm using a pre-cooled cryostat (Leicca, USA). These sections were subsequently placed onto the Visium 10x Genomics slide (10X Genomics Visium Spatial Gene Expression Reagent Kits, PN-1000184), with dimensions tailored to accommodate the 6.5 mm² oligo-barcoded capture areas. An optimal permeabilization duration of 18 min was determined and selected for use. The experimental slide containing the spinal cord tissue was then fixed and subjected to hematoxylin and eosin (H&E) staining. Imaging of the array was undertaken using a Leica DM5500 B microscope (Olympus, Japan), allowing for the comprehensive capture of the entire array and subsequent image merging.

The processing of sequence libraries followed the stipulated guidelines provided by the 10x Genomics Visium Spatial Transcriptomic. The generated data were subjected to analysis using ABI 7500 Software 2.3, contributing to the comprehensive exploration of the spatial transcriptomic landscape. The analysis encompassed the utilization of the Space Ranger software (version 1.0.0) and Seurat (version 4.3.0) package [15]. As a first step, data normalization was executed utilizing sctransform. This normalization procedure aptly accounted for variability in sequencing depth across data points, and concurrently identified high-variance features. To discern the distribution of the designated cell type, the function AddModuleScore from the Seurat package was invoked [20]. This encompassed a pivotal aspect of the analytical process, enabling insights into the composition and spatial distribution of specific cell types within the examined tissue. The marker genes used for spatial localization are listed in Table S1. SpatialFeaturePlot function was used to display genes expression levels. CellChat package was used to construct the cell-cell communication network. Unless otherwise indicated, default parameters were used for all analyses.

### Spatial metabolomics

Spatial metabolomics analysis was conducted using AFADESI-MSI (Ambient Fluidic-Aerosol Desorption Electrospray Ionization Mass Spectrometry Imaging) [21]. This method was executed in both positive and negative-ion modes, facilitated by a Q-Exactive mass spectrometer (Thermo Fisher Scientific, USA). The analysis spanned an m/z range of 100–1,000, maintaining a nominal mass resolution of 70,000. The spray solvent employed was a blend of acetonitrile and water (80:20, V/V), dispensed at a flow rate of 5 mL/min. For positive-ion mode, the sprayer and transport tube voltages were set at 7,500 V and 2,000 V, respectively. Conversely, in negative-ion mode, these voltages were configured as −5,500 V and − 1,500 V. The extracting gas flow was maintained at 45 L/min, with the capillary temperature set at 350 °C.

For analysis, the.raw files were converted into.imzML format. The MSiReader software (version 1.02) was used to simple visualization of molecular distributions [22]. The tolerance was set to 10 ppm and the jet colormap was selected. Other parameters were left as defaults. In order to combine the results of ST and SM, the middle of the three adjacent spinal cord sections was used for ST and the other two were used for SM (ST requires only one section, whereas SM requires two sections). Therefore, the precise localization and characterization of ST and SM were matched. After obtaining the localization information of genes, we checked the corresponding area by MSiReader software and input into R software by Cardinal package. Finally, significantly enriched metabolites (adjusted P value < 0.05) in this region were obtained. Among the candidate metabolites, we identified potential metabolites based on a review of the literature.

### Statistical analysis

Statistical analyses were performed using R (version 4.3.0). Based on prior experience, the scRNA-seq, ST, and SM data included three biological replicates per group. Both in vitro and in vivo experiments comprised six biological replicates per group. Quantitative data are presented as mean ± standard error of the mean (SEM). The specific hypothesis testing methods are as follows: For two-group comparisons, a t-test (for normally distributed data with equal variances), Welch’s t-test (for normally distributed data with unequal variances), or Wilcoxon rank-sum test (for non-normally distributed data, a non-parametric test) was used. For multiple-group comparisons, a one-way ANOVA (for normally distributed data with equal variances), Welch’s one-way ANOVA (for normally distributed data with unequal variances), or Kruskal-Wallis test (for non-normally distributed data, a non-parametric test) was used.

## Results

### Single-cell transcriptomics, spatial transcriptomics and spatial metabolomics profiles in spinal cord injury

**Fig. 1:**
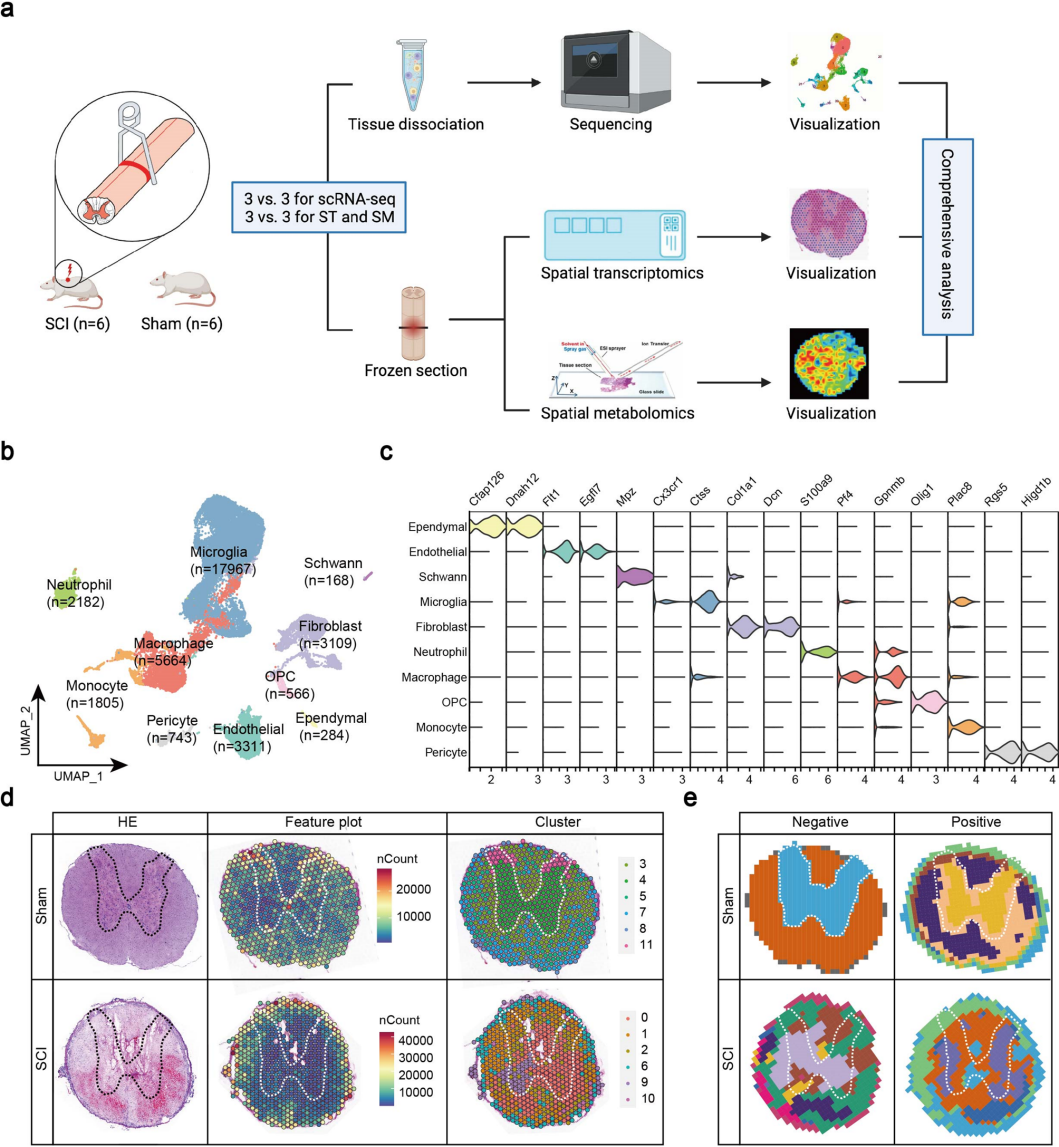
ScRNA-seq, spatial transcriptomics and spatial metabolomics uncover the molecular change after SCI. (**a**) Workflow of this study. In short, spinal cord tissues from 3 SCI and 3 Sham rats were collected for scRNA-seq and 6 other samples (3 SCI and 3 Sham) used for ST and SM. (**b**) UMAP plot presenting main cell types in spinal cord. The x and y axis represents the first and second principal components of UMAP dimensionality reduction, respectively. Each point in the panel represents a cell. Numbers represent number of cells. (**c**) Violin plot of marker genes in each cell type. The color represents the different cell types. (**d**) Representative maps of spatial transcriptomics including HE, feature and cluster plots. HE staining revealed changes after SCI, including occurrence of internal bleeding and tissue degeneration. Feature plot shows the number of reads in each spot. Based on gene expression, capture spots of ST were clustered and 12 clusters were showing. (**e**) Representative maps of spatial metabolomics including negative and positive ion modes. The color represents the different metabolic expression patterns. The same color indicates that the characteristic metabolites in the spot are similar. The dashed line in d and e represents the gray matter of the spinal cord

To assess the cellular and spatial heterogeneities of SCI, we conducted scRNA-seq, ST, and SM (Fig. 1a). After standardizing the quality control process, the median number of genes detected per cell in the scRNA-seq data was 1,977, and the average proportion of mitochondrial UMIs per cell ranged from 4.20% to 5.90%. These results indicate that the data quality control was satisfactory (Figure S1a-d). A total of 35,799 cells from uninjured and injured spinal cord were obtained and clustered into 10 main cell types, including 17,967 microglia, 5,664 macrophages, 3,109 fibroblasts, 3,311 endothelial cells, 1,805 monocytes, 2,182 neutrophils, 743 pericytes, 566 oligodendrocyte progenitor cells (OPCs), 284 ependymal cells, 168 schwann cells (Fig. 1b). These cell types were identified using several widely accepted cell markers (Fig. 1c; Figure S1e). The top 100 marker genes of each cell type are listed in Table S2.

Additionally, to generate unbiased transcriptomic and metabolomic maps of spinal cord before and after injury, ST based on Visium (10X Genomics) platform and SM based on mass spectrometry imaging (MSI) were performed. In the ST data, the average number of genes detected per spot was 2,096, and the proportion of mitochondrial genes per spot ranged from 7.94% to 14.48%, indicating satisfactory data quality control. After data normalization, uniform manifold approximation and projection (UMAP) analysis was performed for dimension reduction and visualization of the clusters. As shown in Fig. 1d-e, the injured spinal cord exhibits transcriptional and metabolic characteristics that are completely different from those in the Sham group (Fig. 1d-e) or normal spinal cord (Figure S2). Moreover, we found that diverse transcriptional and metabolic expression patterns also exist between white matter and gray matter in the normal spinal cord.

### Molecular and Spatial characteristics of microglia populations in spinal cord injury

**Fig. 2:**
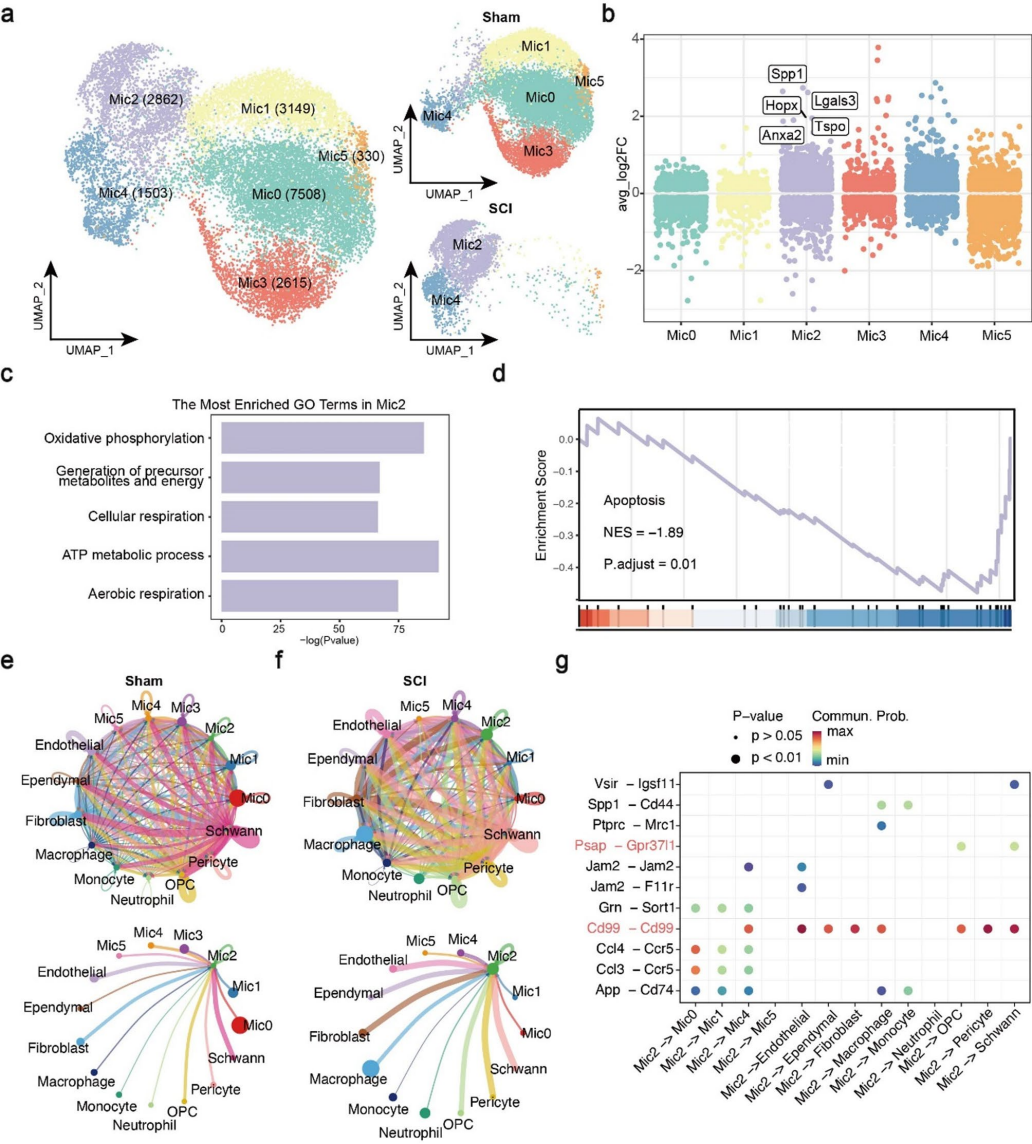
Molecular identification of microglia populations in spinal cord injury. (**a**) UMAP plots showing six microglia subsets and the distribution in two groups. Numbers represent number of cells. (**b**) Scatter plot showing the distribution of marker genes of 6 microglia subsets and top 5 genes of Mic2 were marked. The color represents the different microglia subsets. (c and d) Results of enrichment analysis. Top 5 enriched GO (c) terms and GSEA (d) term of Mic2. (**e**) Results of ligand–receptor analysis in Sham group. For clarity and brevity, the relationship between Mic2 and other cell types was presented separately. (**f**) Results of ligand–receptor analysis in SCI group. (**g**) Dot plot of the interaction scores between Mic2 and others in SCI group. Size of the dot indicates P value. Color of dot indicates interaction score where dark red dots signify stronger predicted interactions

In recent years, the role of microglia in spinal cord injury has garnered increasing attention, particularly for their contributions to inflammation regulation and injury repair processes [12, 23]. For instance, a study demonstrated that microglia facilitate scar-free repair after spinal cord injury in neonatal mice [12]. To better understand the microglia heterogeneity, we subclustered it and obtained 6 microglia subsets, among them four subsets (Mic0, Mic1, Mic3, and Mic5) mainly in Sham group and two types (Mic2 and Mic4) in SCI group (Fig. 2a). Based on known cell markers [11], Mic0, Mic1, Mic3 and Mic5 subsets mainly expressed homeostatic microglia-related markers such as Tmem119 and performed corresponding cellular functions (Figure S3a, b). Mic4 subset was annotated as dividing microglia because it expressed cell cycle-related genes such as Cdk1 and enriched in cell cycle-related pathways (Figure S3a, b). Finally, Mic2 subset expressed migrating microglia-related markers (Igf1 and Msr1), but its enriched GO terms were similar to those of dividing microglia (Figure S3a; Fig. 2c). Therefore, it was considered as intermediate cell type both have the biological functions of dividing and migrating. Notably, Mic2 subset was the main microglia subset after SCI, which highly expressed Spp1, Hopx, Lgals3, Anxa2 and Tspo (Fig. 2b). These genes were enriched in wound healing, positive regulation of cell adhesion, and other processes, indicating anti-inflammation and regeneration functions (Fig. 2c, d) [12]. The top 150 marker genes for each microglia subset and the top 60 differentially expressed genes (DEGs) between SCI and Sham groups are presented in Table S3 and Table S4, respectively. Heatmaps were used to show the expression levels of DEGs between microglia clusters and between SCI and Sham (Figure S4).

Furthermore, ligand–receptor analysis was performed to deeply uncover the function of Mic2 subset (Fig. 2e, f). In contrast, the interactions between Mic2 and endothelial cells, ependymal cells, fibroblasts, OPCs, pericytes, and schwann cells were enhanced after SCI. In detail, interaction plot showed the Cd99-Cd99 interaction was the most enriched ligand–receptor pair in SCI group (Fig. 2g, Figure S3e). This interaction was involved in cell migration and adhesion [24], indicating that Mic2 subset could interact with vascular cells (endothelial, fibroblasts, pericyte) and may involve in angiogenesis. In addition, the Psap-Gpr37l1 interaction was observed between Mic2 and OPC, schwann (Fig. 2g, Figure S3e), which could trigger endocytosis or pro-survival signaling pathways [25, 26]. Moreover, a study reported that the Psap-Gpr37l1 interaction provided neuroprotection and glioprotection within nervous system [27]. The violin plots showed the expression levels of ligands and receptors in each cell type (Figure S3c-d). Moreover, based on preliminary studies, ligand–receptor analysis was performed in ST data. The results showed that Mic2 could promote angiogenesis via Ptn-Ncl interation (Figure S5). In summary, Mic2 showed a potential protective effect against SCI.

**Fig. 3:**
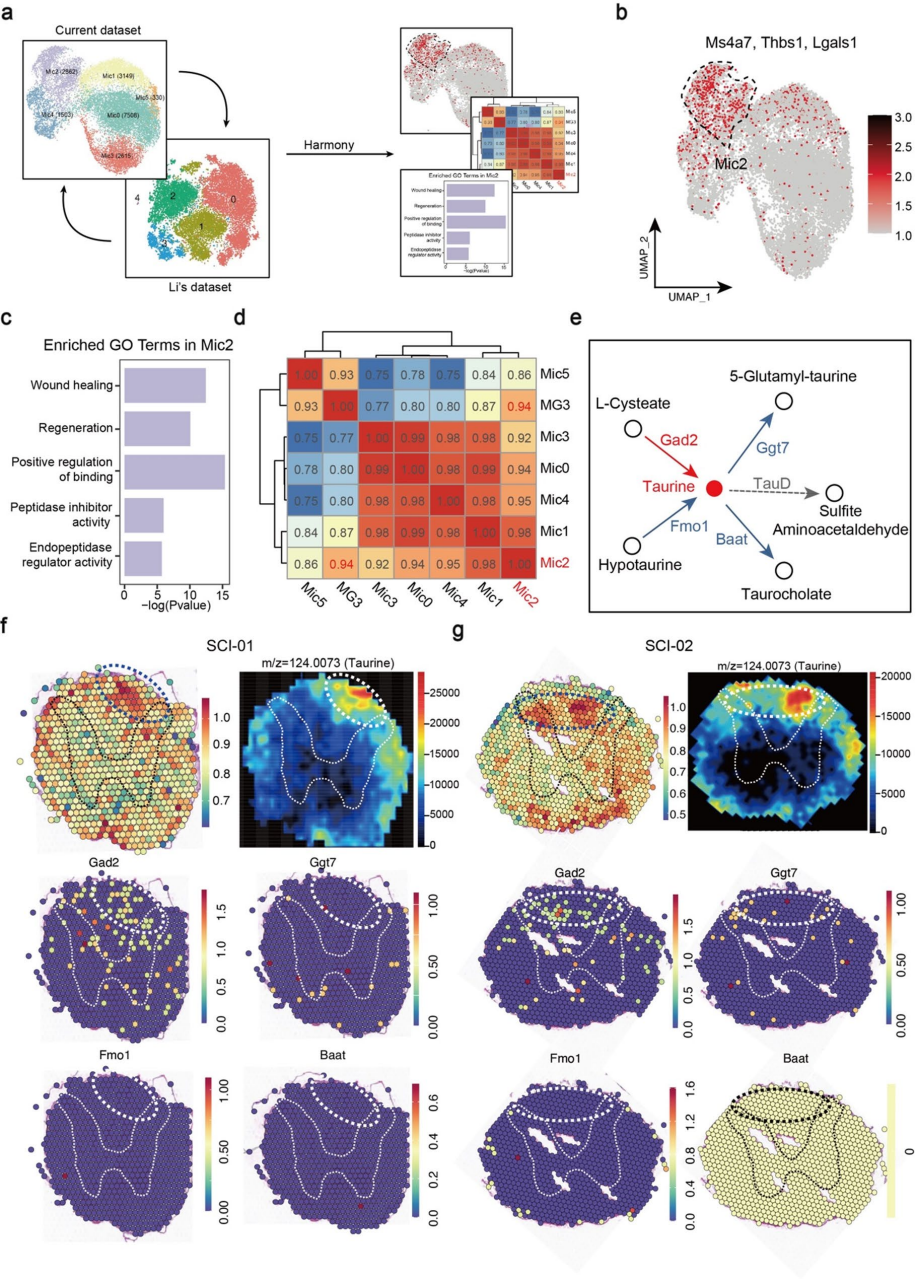
Focused analyses of regeneration-promoting microglia. (**a**) Overview of the integrated analysis method. Briefly, harmony algorithm was used to integrate the our current scRNA-seq dataset and Li’s dataset. (**b**) Feature plot showing the average expression of MG3 markers (Ms4a7, Thbs1 and Lgals1) in microglia subsets from this study. These markers mainly expressed in Mic2. (**c**) Regeneration-related GO terms are enriched in Mic2. (**d**) Clustering heatmap based on Pearson’s correlation coefficient between MG3 (Li’s dataset) and microglia subsets (current dataset). It reveals the highest correlation between MG3 and Mic2. (**e**) A schematic showing upstream metabolites, downstream metabolites and related-enzymes of taurine metabolism. Colors indicate upregulation and downregulation of metabolites and enzymes: blue represents down-regulation and red represents up-regulation. (**f** and **g**) Comprehensive analysis including scRNA-seq, spatial transcriptomics and spatial metabolomics of Mic2 subset in SCI-01 (f) and SCI-02 (g) samples. We circled the spinal cord gray matter for localization of spinal cord tissue. For both ST and SM maps, the color indicates the relative expression level of metabolites or genes, with red indicating higher level while blue indicating lower level of expression

Recent studies have reported the existence of a microglial population with regenerative properties that can facilitate spinal cord regeneration following SCI in both neonatal and adult spinal cords [12, 28]. In this study, we reanalyzed the published dataset [12] found regeneration-promoting microglial genes (Ms4a7, Thbs1, and Lgals1) expression was concentrated in Mic2 (Fig. 3a, b; Figure S6a, b). Further functional enrichment and correlation analyses revealed Mic2 subset, highly correlated with MG3 (regeneration-promoting microglia), along with enrichment in repair-related pathways, notably those pertaining to wound healing and regeneration (Fig. 3c, d).

To further explore the spatial molecular characteristics of Mic2 subset, “AddModuleScore” was applied to map the Mic2 subset in ST images, revealing that it was mainly distributed in the white matter, especially in the dorsal region of injured spinal cord (Fig. 3f, g; Figure S6c). After determining the localization of Mic2, SM analysis revealed that the Mic2-populated area was characterized by a high distribution of taurine, an organic acid known to promote axonal regeneration after complete SCI (Table S5). Interestingly, we observed a tendency for the Mic2 subset to aggregate into clusters. To investigate taurine metabolism in the Mic2 subset, we systematically searched the literature and taurine metabolism–related databases (KEGG, HMDB) to identify enzymes and transporters directly involved in the upstream and downstream metabolic processes of taurine. These genes were selected as representative markers because they occupy critical positions in taurine metabolic pathways. We further analyzed the expression levels of taurine metabolism related-enzymes and found that Gad2 was at a high expression level and Fmo1, Baat, Gpt7 was at low expression levels (Fig. 3f, g). Finally, we summarized the metabolism pathway, as shown in Fig. 3e. Among taurine metabolism related-enzymes, TauD was not detected in our ST data, so it was not displayed. Based on these results, we speculate that high level of Gad2 led to accumulation of taurine. Together, our results suggested that injury-induced Mic2 distributed in white matter of dorsal, exhibited elevated taurine expression, and had the potential to promote spinal cord repair.

### Integrating analysis reveals molecular changes and Spatial signature in macrophage subsets

**Fig. 4:**
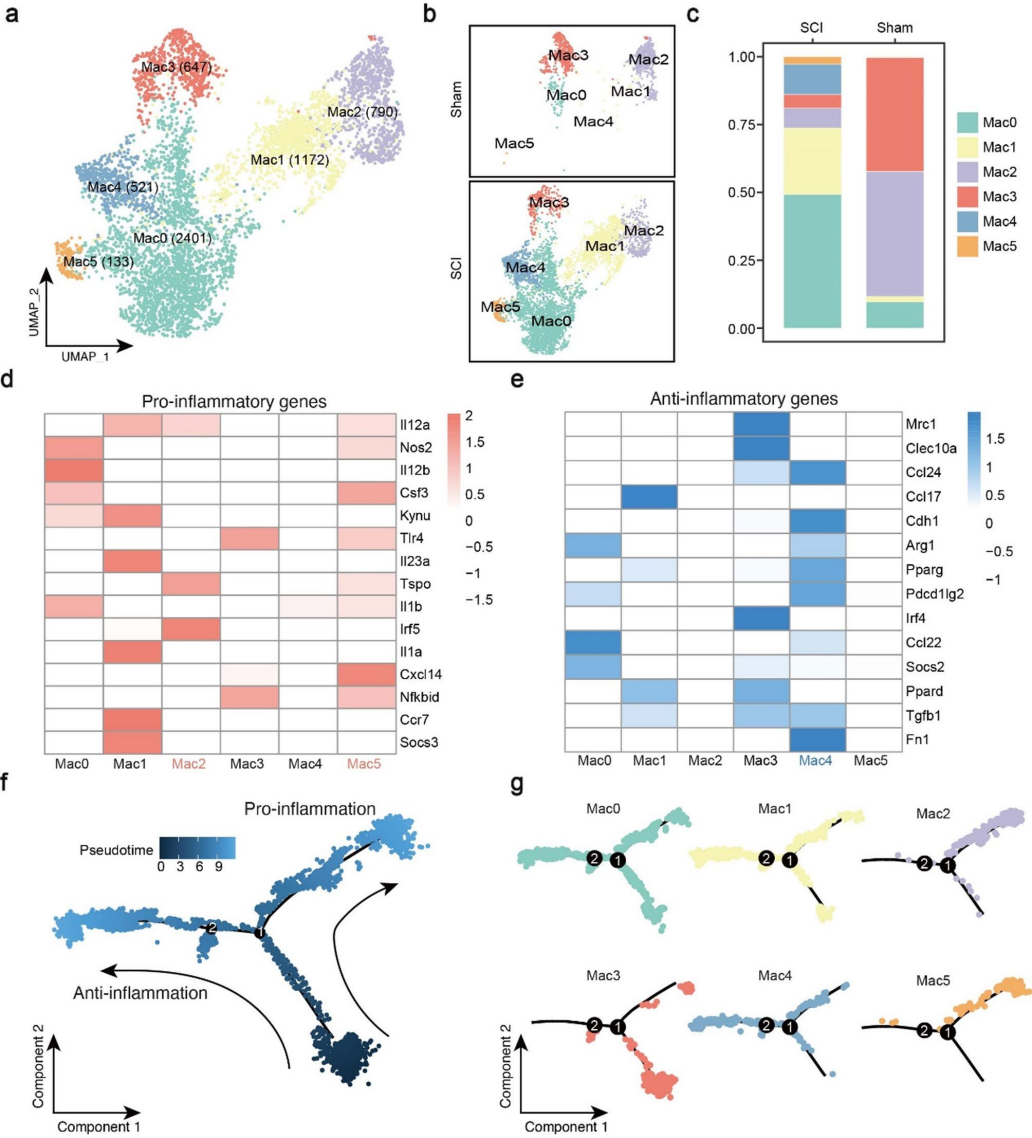
Integrating analysis reveals molecular changes in macrophage subsets. (**a**) UMAP plot showing six macrophage subsets. The number of cells of each subset was indicated in the figure. (**b** and **c**) UMAP plot and accumulative bar diagram showing the distribution of six macrophage subsets in two groups. (**d** and **e**) Heatmap of pro-inflammatory genes and anti-inflammatory genes expression among all macrophage subsets. Lighter colors reflect lower expression levels, while darker ones indicate higher expression levels. (**f** and **g**) Pseudotime analysis depicting the evolution of six macrophage subsets. Two main branch trajectories were identified (**f**). The distribution of each subset in pseudotime trajectory was presented (**g**). Each point represents one cell

The current data revealed that macrophages were the second most abundant cell type. Among these, six distinct macrophage subsets were identified, with the majority (Mac0, Mac1, Mac4, Mac5) emerging after SCI (Fig. 4a-c). Firstly, the heatmaps showed the expression levels of pro-inflammatory and anti-inflammatory genes [11] in different macrophage subsets (Fig. 4d, e). Specifically, Mac2 and Mac5 highly expressed pro-inflammatory genes, while Mac4 expressed anti-inflammatory genes. Notably, Mac0, Mac1 and Mac3 both expressed pro-inflammatory and anti-inflammatory genes and were not considered as classical M1/M2 subsets. Interestingly, the marker genes of Mac0, Mac1, and Mac3 were mainly enriched in cellular detoxification, cellular response to stress and immune related pathways, respectively (Figure S7a). Moreover, the established M1/M2 gene sets were used to calculate the score to confirme the state of each macrophage subset. The results showed that Mac2 and Mac5 have high M1 score and Mac4 has the high M2 score (Figure S7d). Finally, the top150 marker genes of each subset were showed in Table S6.

**Fig. 5:**
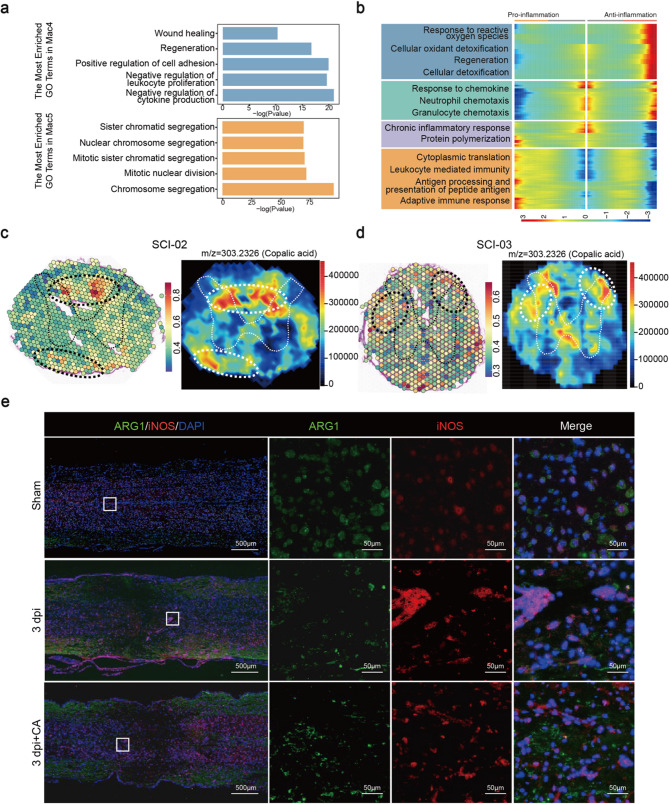
Focused analyses showing the multi-omics of macrophage subsets. (**a**) Top 5 enriched GO terms in Mac4 and Mac5, respectively. (**b**) BEAM analysis indicating different expression patterns between pro-inflammation and anti-inflammation macrophage subsets. (**c** and **d**) Comprehensive analysis including scRNA-seq, spatial transcriptomics and spatial metabolomics of Mac2 subset in SCI-02 (c) and SCI-03 (d) samples. We circled the spinal cord gray matter for localization of spinal cord tissue. For both ST and SM maps, the color indicates the relative expression level of metabolites or genes, with red indicating higher level while blue indicating lower level of expression. (**e**) Immunohistochemistry results reveal that ARG1 levels increase and iNOS levels decrease following treatment with copalic acid (CA) at 3 day post-injury (dpi). Each group had six replicates

To investigate the polarization process of the macrophage subsets, pseudotime analysis was performed. There were two main branch trajectories and the arrows indicated the direction of differentiation (Fig. 4f). As anticipated, we observed that Mac2 and Mac5 distributed in “pro-inflammatory” branch while Mac4 was in another branch (Fig. 5g). Along the trajectory, pro-inflammatory genes such as Apoc1 and S100a11 were expressed in a sustained high level during the “pro-inflammation” branch and anti-inflammatory genes (Anxa3, Stmn1) gradually increased along another branch (Figure S7b; Fig. 5a).

We further clustered all genes along the trajectory and identified 4 expressed patterns (Fig. 5b). Functional enrichment analysis showed that genes with spinal cord repair function such as cellular detoxification and regeneration had a high expression level in “anti-inflammatory” branch. In contrast, we identified some immune associated pathway such as leukocyte mediated immunity and antigen processing and presentation of peptide antigen in “pro-inflammation” branch. Furthermore, we observed that the other two expression patterns are primarily enriched in inflammation-related pathways, such as response to chemokine, neutrophil chemotaxis and chronic inflammatory response. This may be due to the presence of several macrophage subsets (Mac0, Mac1 and Mac3) in our data that co-express both pro-inflammatory and anti-inflammatory genes. The results of enrichment analysis align consistently with the aforementioned findings (Fig. 5a).

The anti-inflammatory macrophage subset played a key role in SCI repair [29], so we further analyzed the spatial molecular characteristics of Mac4 subset. Similar to Mic2, we also found the Mac4 subset aggregated into clusters and scattered in the transverse section of the spinal cord (Fig. 5c, d; Figure S7c). SM analysis showed Mic2-populated area was characterized by high distribution of copalic acid (CA) (Fig. 5c, d; Figure S7c; Table S7). CA presented anti-inflammatory, antimicrobial, antiparasitic, cytotoxic, chemopreventive, antimutagenic, and antigenotoxic activities, without adequately studied [30, 31]. As a consequence, we proceeded with in vitro and in vivo studies to validate its anti-inflammatory function. BV-2 represents central nervous system-resident macrophages and RAW264.7 acts as monocyte-derived macrophages. The results showed the expression levels of pro-inflammation genes (CD86, CD80, IL-1β) confirmed by qPCR were decreased after CA treatment (Figure S8a). Moreover, CA can suppress inflammatory responses in mice after SCI at 1, 3, and 7 dpi. Immunohistochemistry results reveal that ARG1 levels increase and iNOS levels decrease following treatment with CA (Fig. 5e, Figure S8b).

### Single cell and Spatial multi-omics reveal a new regeneration repair-promoting fibroblast subset

**Fig. 6:**
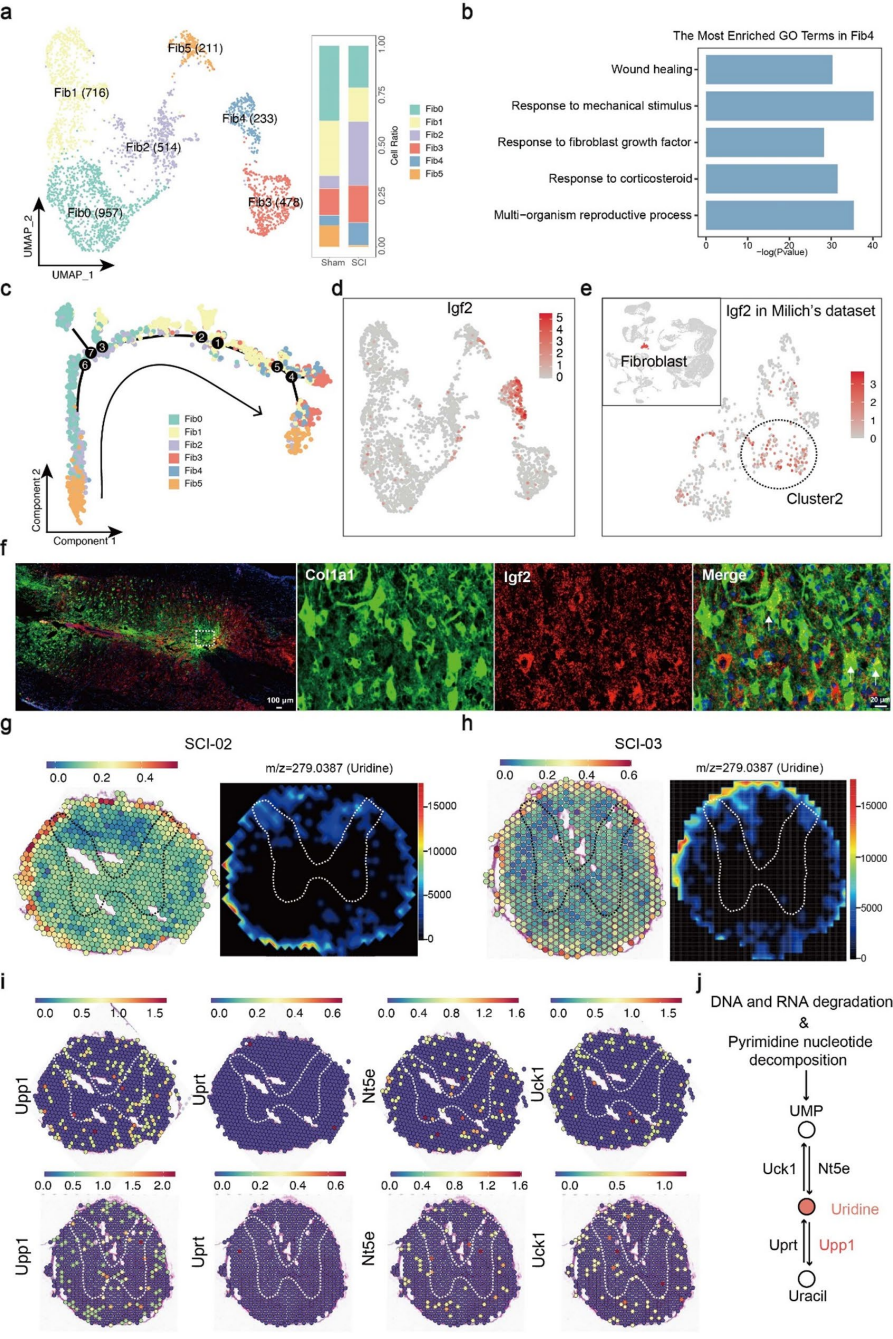
Single cell and spatial multi-omics reveal a new regeneration-promoting fibroblast subset. (**a**) UMAP plots showing six fibroblast subsets and the distribution in two groups. Numbers represent number of cells. (**b**) Spinal cord repair related GO terms enriching in Fib4. (**c**) Results of pseudotime analysis. Fib4 mainly distributed in the end of pseudotime trajectory. (**d**) Igf2 was the marker gene of regeneration-promoting fibroblast subset. The color indicates the relative expression level of igf2: darker colors indicate higher expression levels. (**e**) The regeneration-promoting fibroblast subset was also found in Milich’s dataset. (**f**) The results of immunofluorescence staining. Col1a1 is the marker gene of fibroblasts and Igf2 represents the Fib4. (**g-i**) Comprehensive analysis including scRNA-seq, spatial transcriptomics and spatial metabolomics of Fib4 subset in SCI-02 (g) and SCI-01 (h) samples. We circled the spinal cord gray matter for localization of spinal cord tissue. For both ST and SM maps, the color indicates the relative expression level of metabolites or genes, with red indicating higher level while blue indicating lower level of expression. (**j**) A schematic showing upstream metabolites, downstream metabolites and related-enzymes of uridine metabolism. Red represents up-regulation of metabolite and enzyme

Traditionally, fibroblasts have been primarily associated with the formation of fibrous scars following SCI, which could impede the process of regeneration [32]. In the present study, the fibroblasts was clustered into 6 subsets (Fig. 6a), and top10 marker genes of each subset were shown in the heatmap (Figure S9a). Furthermore, top150 marker genes of each subset were shown in Table S8. As shown in Figure S6b, Fib0, Fib2, Fib3 were enriched in response to SCI injury. Fib0, Fib2, Fib5 were enriched in inflammation-relative pathways and Fib0, Fib1 were enriched in fibrous scar formation (Figure S9b; Fig. 6c). Notably, Fib4 subset, expressing high level of Igf2 was identified with repair-promoting function (Fig. 6b, d). To corroborate the presence of the Fib4 subset following SCI, we re-analyzed single-cell data from another study [11] and confirmed existence of Fib4 (Fig. 6e). On the other hand, immunofluorescence staining was performed (Fig. 6f) for validation. Igf2 is a secreted protein and we found it expressed in a portion of Fibroblasts (Fig. 6f).

To further explored the spatial molecular characteristics of Fib4 subset, ST and SM analyses were performed. We noticed that Fib4 subset distinctly located around surface of the injured spinal cord (Fig. 6g, h; Figure S9c). Furthermore, SM analysis showed Fib4-populated area was characterized by high distribution of uridine, a pyrimidine nucleoside, which can rejuvenate aged human stem cells and promote regeneration of various tissues in vivo (Table S9) [33]. Finally, we showed the expression level of related enzymes in Fig. 6i and Figure S9d, and summarized the metabolism pathway showing in Fig. 6j. Among four enzymes, Upp1, Nt5e, and Uck1 were at a high level and others were at low levels (Fig. 6i). In the Fib4 fibroblast subtype, we observed concurrent high expression of key uridine metabolism–related genes, including Upp1, Nt5e, and Uck1. Upp1 catalyzes the reversible phosphorolysis of uridine, Nt5e contributes to nucleotide catabolism by converting nucleotides into nucleosides, and Uck1 phosphorylates uridine to UMP for nucleotide salvage. The coordinated upregulation of these genes suggests that Fib4 cells maintain a dynamic uridine metabolic state, balancing degradation and salvage pathways. Such metabolic flexibility may support the energetic and biosynthetic demands required for tissue repair and remodeling after spinal cord injury, highlighting the potential functional significance of Fib4 fibroblasts in the injury response.

**Fig. 7:**
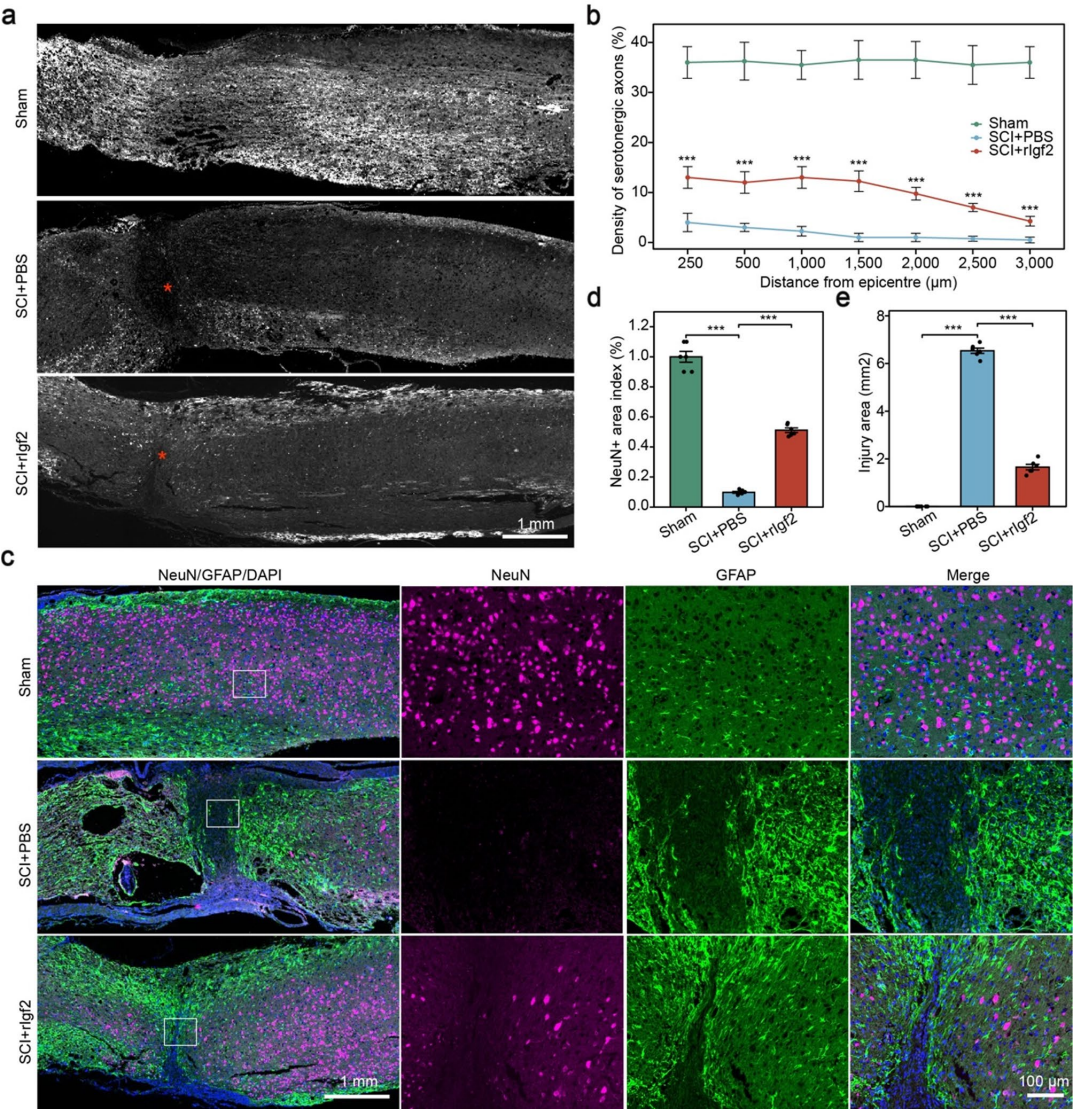
Recombinant Igf2 (rIgf2) promotes neuroprotection and axon regeneration after spinal cord injury. (**a**) Representative 5HT-stained axons in the injury zone. Scale bar: 1 mm. Red stars indicate the lesion site. (**b**) Density of serotonergic (5HT) axons at varying distances from the injury epicenter. (**c**) Representative images of spinal cord sections stained for NeuN (neurons, red), GFAP (astrocytes, green), and DAPI (nuclei, blue) at 28 days post-injury. Scale bars: 1 mm (overview), 100 μm (insets). (**d**) Quantification of NeuN + area index. (**e**) Lesion area measurement. Each group had six replicates. *** *P* < 0.001

Our single-cell analysis identified a protective Igf2 + fibroblast subset in SCI, suggesting that Igf2 secretion may mediate neuroprotection. To validate this, we administered recombinant Igf2 (rIgf2) following SCI and evaluated outcomes at 28 dpi. The SCI + rIgf2 group showed significantly higher density of serotonergic axons (5HT) near the injury epicenter compared to the SCI + PBS control. Axon density decreased with distance from the epicenter but remained elevated in rIgf2-treated animals (Fig. 7a, b). Immunofluorescence staining revealed that rIgf2 treatment preserved NeuN + neurons compared to PBS controls. The NeuN + area index was significantly higher in the SCI + rIgg2 group, indicating enhanced neuronal survival (Fig. 7c, d). Finally, the injury area was markedly smaller in rIgf2-treated mice versus PBS controls, suggesting rIgf2 mitigated tissue damage (Fig. 7e). Moreover, to assess functional recovery after SCI, we performed longitudinal behavioral tests in three groups over 28 dpi. The SCI + rIgf2 group showed significant improvement in hindlimb function compared to the SCI + PBS group (Figure S10a). The maximum incline angle was significantly higher in the SCI + rIgf2 group vs. SCI + PBS, indicating improved limb strength and coordination (Figure S10b). The relative intensity of hind paw strikes was significantly higher in the SCI + rIgf2 group vs. SCI + PBS and rIgf2-treated mice showed faster movement than PBS controls, suggesting better motor control (Figure S10c-f).

## Discussion

Previous studies have deeply explored the transcriptomic landscape after SCI at the single-cell level [11, 28]. However, these studies lacked the spatial resolution to characterize the genes and metabolites. Here, we identified three cell subsets that may promote regeneration after SCI using scRNA-seq, ST, and SM technologies. Among the three cell subsets, Mic2 in microglia and Mac4 in macrophages aggregated into clusters. The Mic2-related cluster was mainly distributed in the white matter of the dorsal region of the injured spinal cord, and this area highly expressed taurine. Unlike Mic2, the Mac4-related cluster was distributed randomly without a fixed pattern, and the corresponding area highly expressed CA. Furthermore, Fib4 in fibroblasts was mainly distributed around the injured spinal cord, and the corresponding area highly expressed uridine.

Previous studies have explored a regeneration-promoting microglia subset in both neonatal and adult SCI mice [12, 28]. This particular microglial subset was capable of secreting fibronectin and binding proteins, which facilitated the formation of extracellular matrix bridges, and it expressed peptidase inhibitors to mitigate inflammation [8]. However, the molecular characteristics and spatial expression of this subset after SCI remained unclear. In our current study, the regeneration-promoting microglia subset was consistently distributed in the white matter, particularly in the dorsal region of the injured spinal cord, which is crucial for the recovery of the posterior funiculus of the spinal cord, associated with deep sensation. This may also explain why sensory recovery is better than motor recovery after SCI. Moreover, we found that taurine was highly expressed in the regeneration-promoting microglia subset-enriched area. Taurine, a sulfur-containing amino acid recognized as semi-essential in mammals [34], has demonstrated its capacity to alleviate injuries induced by inflammation and oxidative stress in various disease models [35–38]. Furthermore, taurine has been reported to mitigate inflammatory responses and promote axonal regeneration post-SCI [39, 40]. Recently, an investigation highlighted the decline of taurine levels during aging and revealed that its supplementation could reverse this decrease, consequently enhancing health span and life span in mice, worms, and monkeys [41]. The collective evidence, including our own findings, strongly suggests that taurine holds promise as a potential therapeutic intervention for central nervous system repair.

The predominant cell type initiating neuroinflammatory reactions after SCI is macrophages, which include both central nervous system-resident and peripheral monocyte-derived populations [42–44]. Although the M1/M2 phenotype classification of macrophages is increasingly being questioned, research on anti-inflammatory macrophages remains meaningful [29]. Our study specifically focused on the anti-inflammatory macrophage subset (Mac4 subset in this study) and found that it aggregated into clusters, similar to the Mic2 subset. Then we identified CA was enriched in Mac4-related area. CA is one of the diterpenoid acids in copaiba oil, with few studies reported on tis effects [31]. Existing research has indicated that CA possessed the capacity to inhibit the chaperone function of α-crystallin and heat shock protein 27kD [31]. In this study, we confirmed the anti-inflammatory activity of CA trough in silicon, in vitro, and in vivo experiments. Together, Mac4 subset would aggregate into cluster and scatter in spinal cord, characterized by high expression of CA. These results enriched our understanding on spatial characteristics of anti-inflammatory macrophages, especially the spatial metabolic characteristic.

It is generally believed that fibroblasts are the main drivers of fibrotic scarring after SCI [45]. Traumatic spinal cord injuries caused fibroblasts to invade lesion sites and form fibrous scarring [46], which was thought to negatively affect disease progression in SCI by blocking progenitor cells from entering the injury core and facilitating axon regeneration; consequently, therapies have been proposed to target these scar to stimulate recovery [47]. However, our investigation has identified a novel subset of fibroblasts with reparative potential, distinguished by heightened expression of Igf2. Insulin-like growth factor 2 (IGF2) emerged as a crucial driver of synaptic plasticity, learning, and memory [48]. These protective effects primarily transpired via the Igf2 receptor (Igf2r), which was extensively expressed in neurons and overseed protein trafficking, synthesis, and degradation [49]. Moreover, we found Fib4-populated area was characterized by high distribution of uridine. Recently, uridine was identified as a potent regeneration promoting factor [33]. In summary, we found a new fibroblast subset. One one hand, it could secrete Igf2 to bind with Igf2r in neurons and play a role in neuronal repair; on the other hand, its downstream metabolite, uridine, possessed a neural repair effect.

The pursuit of functional repair following SCI remains a prominent focus, yet al.so presents a formidable challenge. Diverse strategies, encompassing stem cell, drug, and gene therapies, have been devised, but their efficacy remains suboptimal [7, 50]. Notably, the intrinsic capacity for self-repair following SCI is definitely constrained, including its spatial characteristic including ST and SM remains unclear. For instance, astrocytes can secrete axon growth-supporting proteins that facilitate axon regeneration by promoting layer adhesion [51]. In this present study, we have identified three distinct cell subsets that possess regenerative capabilities, determined their spatial transcriptional and metabolic features enriched within these regions. For example, Mic2, one of microglia subsets, mainly distributed in white matter of dorsal of injured spinal cord and highly expressed taurine, which could promote regeneration. Through this comprehensive investigation, we posit that targeting specific cell subsets along with their distinctive metabolic profiles in particular spatial area should assume a prominent role in the forthcoming phase of therapeutic advancement for SCI treatment.

Although our study offers significant insights, it is essential to acknowledge certain inherent limitations. Firstly, since this study only performed scRNA-seq and did not include single-nucleus RNA sequencing (snRNA-seq), information on neurons was lost, including data on neurons themselves and their interactions with other cell types. We hope that future studies, through the addition of snRNA-seq or more advanced sequencing technologies, will further refine and complement these findings. Moreover, due to technical constraints, scRNA-seq, ST, and SM were performed on separate animals, which may introduce some degree of variability and potential bias. We anticipate that future advancements in single-cell spatial multi-omics technologies for small sample sizes will address this limitation. Finally, this study is primarily descriptive in nature. Although partial in vitro and in vivo experimental validation was conducted, further experimental evidence is required to fully elucidate the underlying mechanisms.

## Conclusions

In this study, we identified three cell subsets (Mic2, Mac4, Fib4) that contribute to spinal cord repair and determined their spatial transcriptional and metabolic features. Among them, Mic2 is predominantly distributed in the white matter, particularly in the dorsal region of the injured spinal cord, and exhibits high expression of taurine. Mac4 and Fib4 exhibit high expression of copalic acid and uridine, respectively. This offers a multi-omics framework for identifying therapeutic targets for SCI.

## Supplementary Information

Supplementary Material 1.

Supplementary Material 2.

## Data Availability

Single cell sequencing, spatial transcriptomics and spatial metabolomics data are available in figshare (10.6084/m9.figshare.28219310).

## Abbreviations

SCI: Spinal cord injury
scRNA-seq: Single-cell RNA sequencing
ST: Spatial transcriptomics
SM: Spatial metabolomics
OPC: Oligodendrocyte progenitor cells
MSI: Mass spectrometry imaging
UMAP: Uniform manifold approximation and projection
CA: Copalic acid
IGF2: Insulin-like growth factor 2
H&E: Hematoxylin and eosin
