# Supplementary material for: Integrating single-cell RNA sequencing and spatial multi-omics reveals the molecular signature of regeneration after spinal cord injury
Source: Biomark Res. 2025 Oct 21;13:130. doi: 10.1186/s40364-025-00845-4 (PMC12542165; doi:10.1186/s40364-025-00845-4)
Supplement: Supplementary file 2 — Supplementary Material 2. [file 40364_2025_845_MOESM2_ESM.docx]

**Supplementary Information for**

Integrating single-cell RNA sequencing and spatial multi-omics reveals the molecular signature of regeneration after spinal cord injury


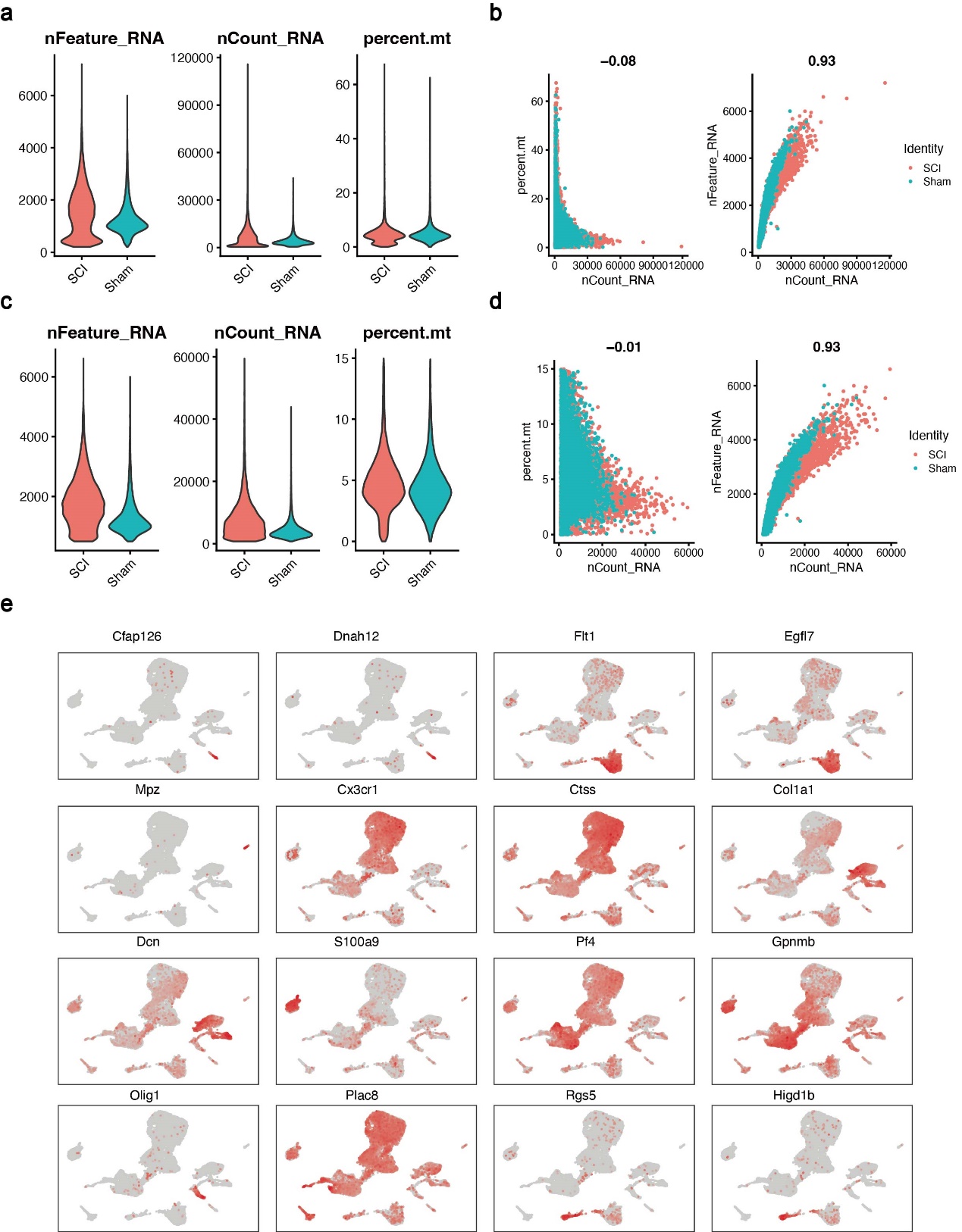


**Figure S1. Quality assessment of single-cell data.**

(**a-d)** Quality control of single-cell data. Violin plots showing the number of feature RNA, count RNA and mitochondrial gene percent before (**a**) and after (**c**) quality control. Scatter plots showing a high correlation coefficient between feature RNA and count RNA (**b**, **d**).

(**e**) Feature plots of selected marker genes. The color indicates the relative expression level of marker genes: darker colors indicate higher expression levels.


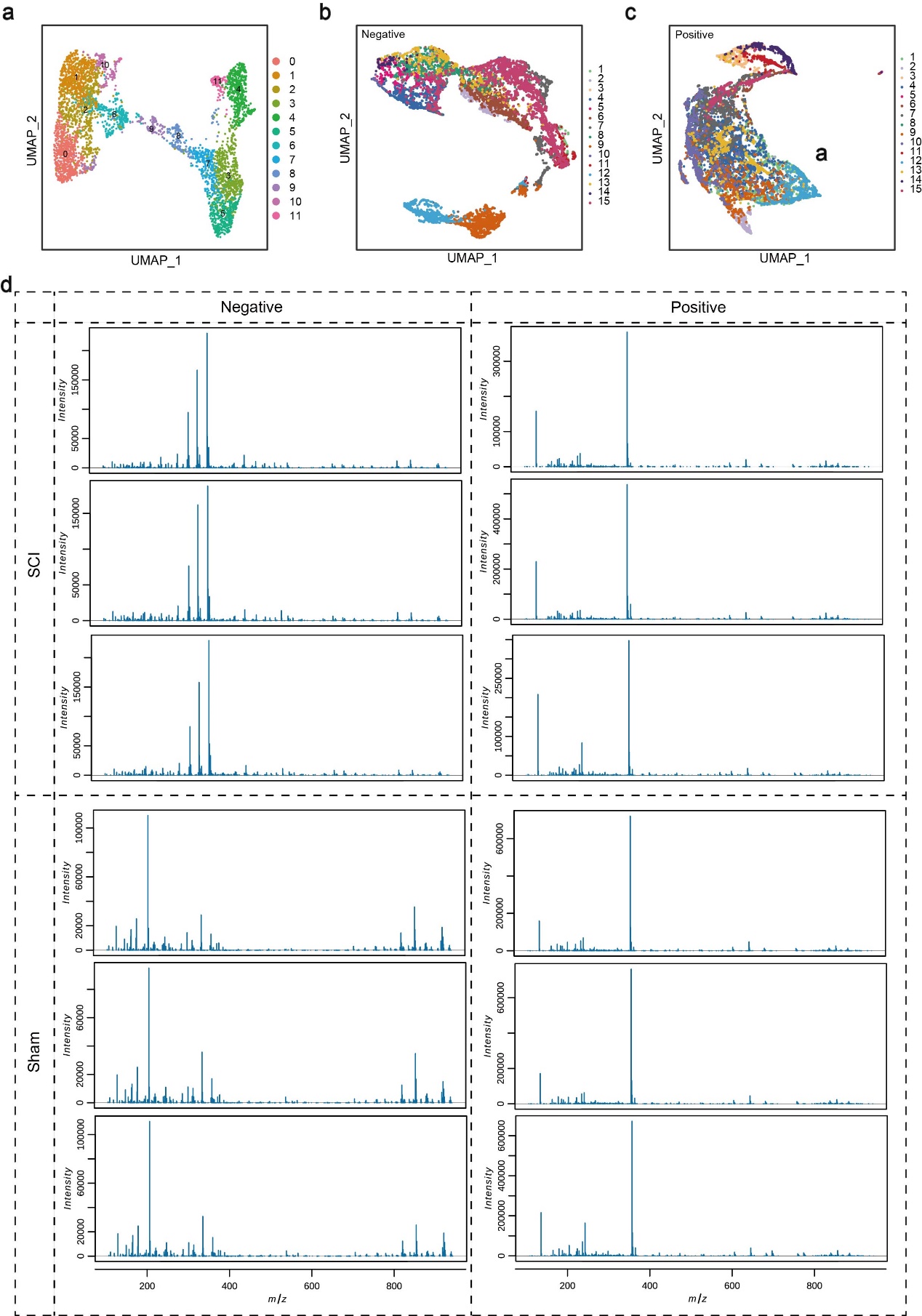


**Figure S2. Quality assessment of ST and SM data.**

(**a**) UMAP plot showing the clusters of ST. Based on gene expression, capture spots of ST were clustered and 12 clusters were showing. Each point represents one spot of ST.

(**b** and **c**) UMAP plots showing the clusters of SM in negative (**b**) and positive (**c**) ion modes, respectively. A total of 15 clusters were showing.

(**d**) Mass spectrograms of all samples containing negative and positive ion modes.


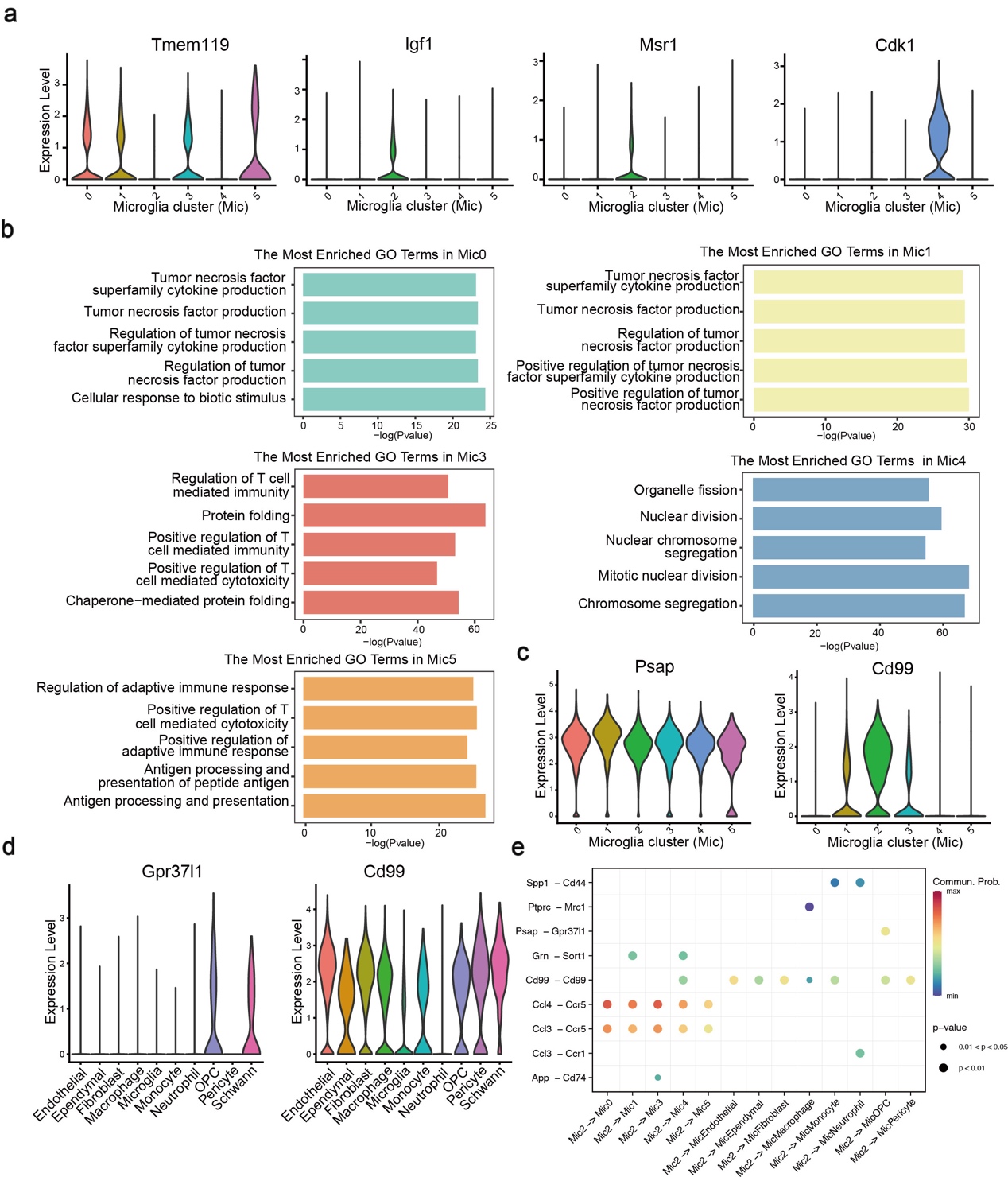


**Figure S3. Molecular characteristics of microglia populations in spinal cord injury.**

(**a**) Violin plots showing the expression level of cell markers in 6 microglia subsets.

(**b**) Top 5 enriched GO terms in Mic0-Mic1 and Mic3-Mic5, respectively.

(**c**) Violin plots showing the expression level of Psap and Cd99 in 6 microglia subsets.

(**d**) Violin plots showing the expression level of Gpr37l1 and Cd99 in each cell type.

(**e**) Dot plot of the interaction scores between Mic2 and others in Sham group. Size of the dot indicates P value. Color of dot indicates interaction score where dark red dots signify stronger predicted interactions.


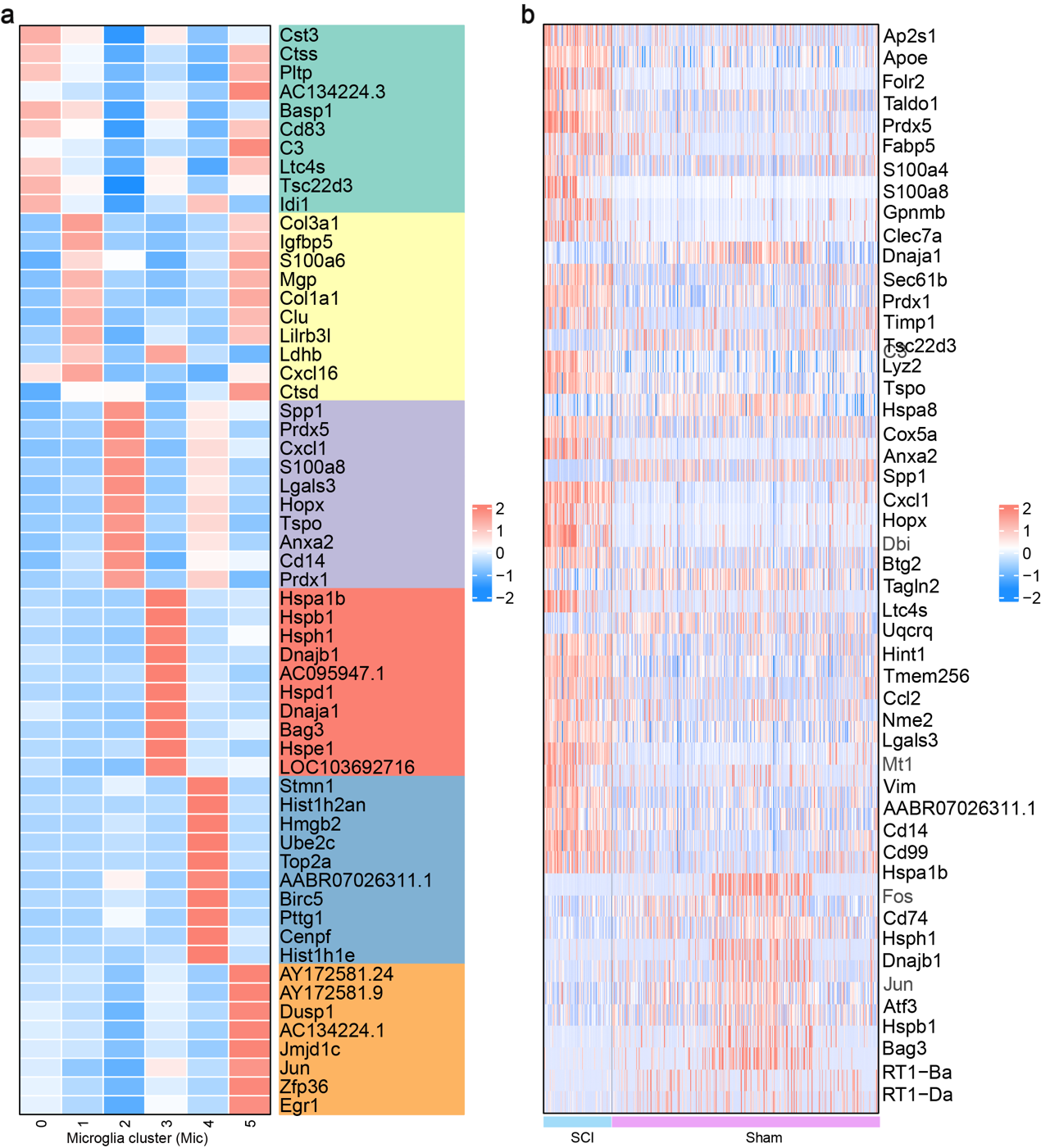


**Figure S4. Differentially expressed genes (DEGs) between microglia clusters and between SCI and Sham.**

(**a**) Heatmap showing top 10 marker genes of 6 fibroblast subsets. The color indicates the relative expression level of marker genes, with red indicating higher level while blue indicating lower level of expression.

(**b**) Heatmap showing top 60 DEGs between SCI and Sham. The color indicates the relative expression level of marker genes, with red indicating higher level while blue indicating lower level of expression.


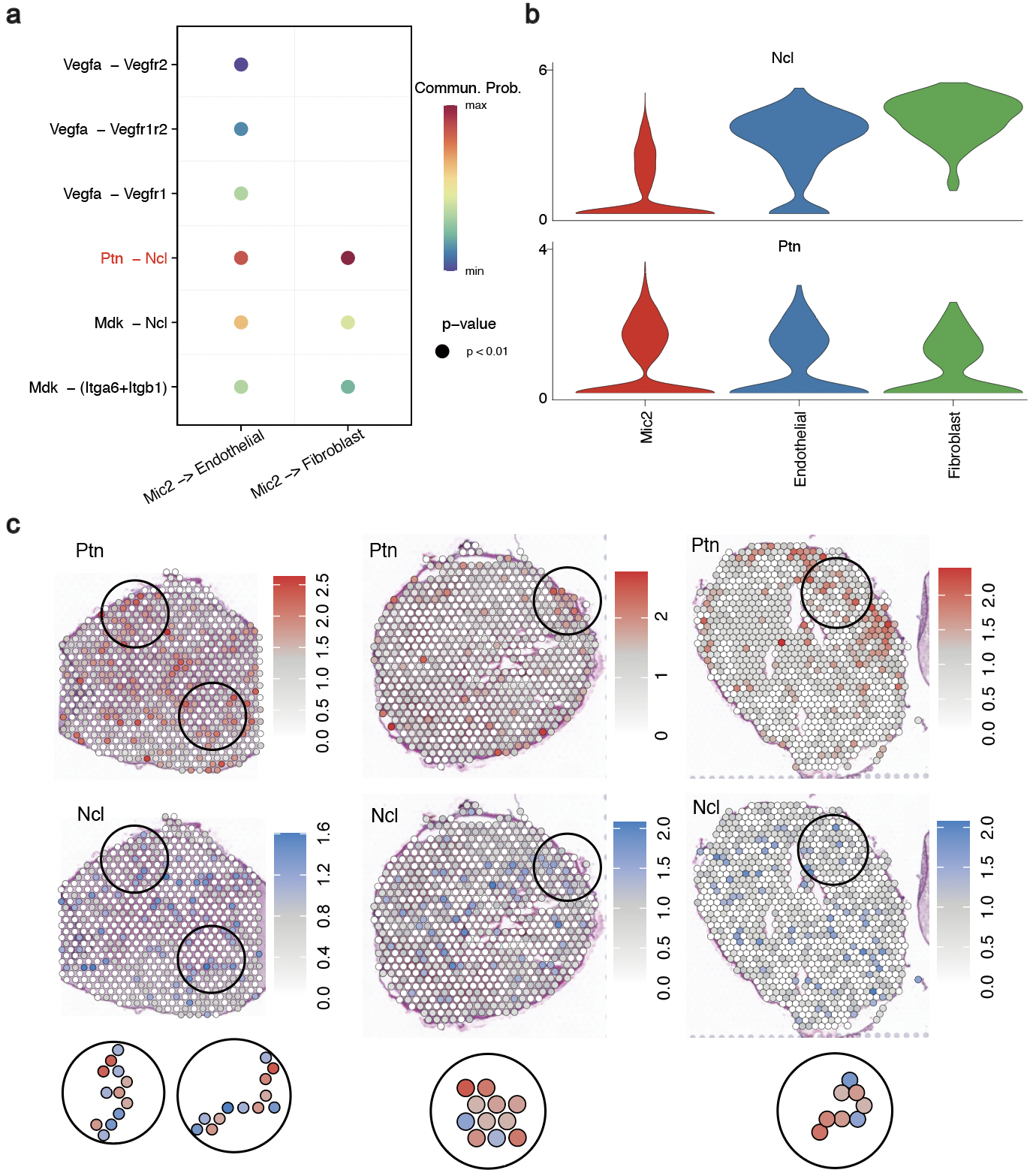


**Figure S5. Results of ligand–receptor analysis in ST.**

(**a**) Dot plot of the interaction scores between Mic2 and endothelial, fibroblast. Size of the dot indicates P value. Color of dot indicates interaction score where dark red dots signify stronger predicted interactions.

(**b**) Violin plots showing the expression level of Ncl and Ptn.

(**c**) The ligand-receptor co-localization analysis in ST.

.


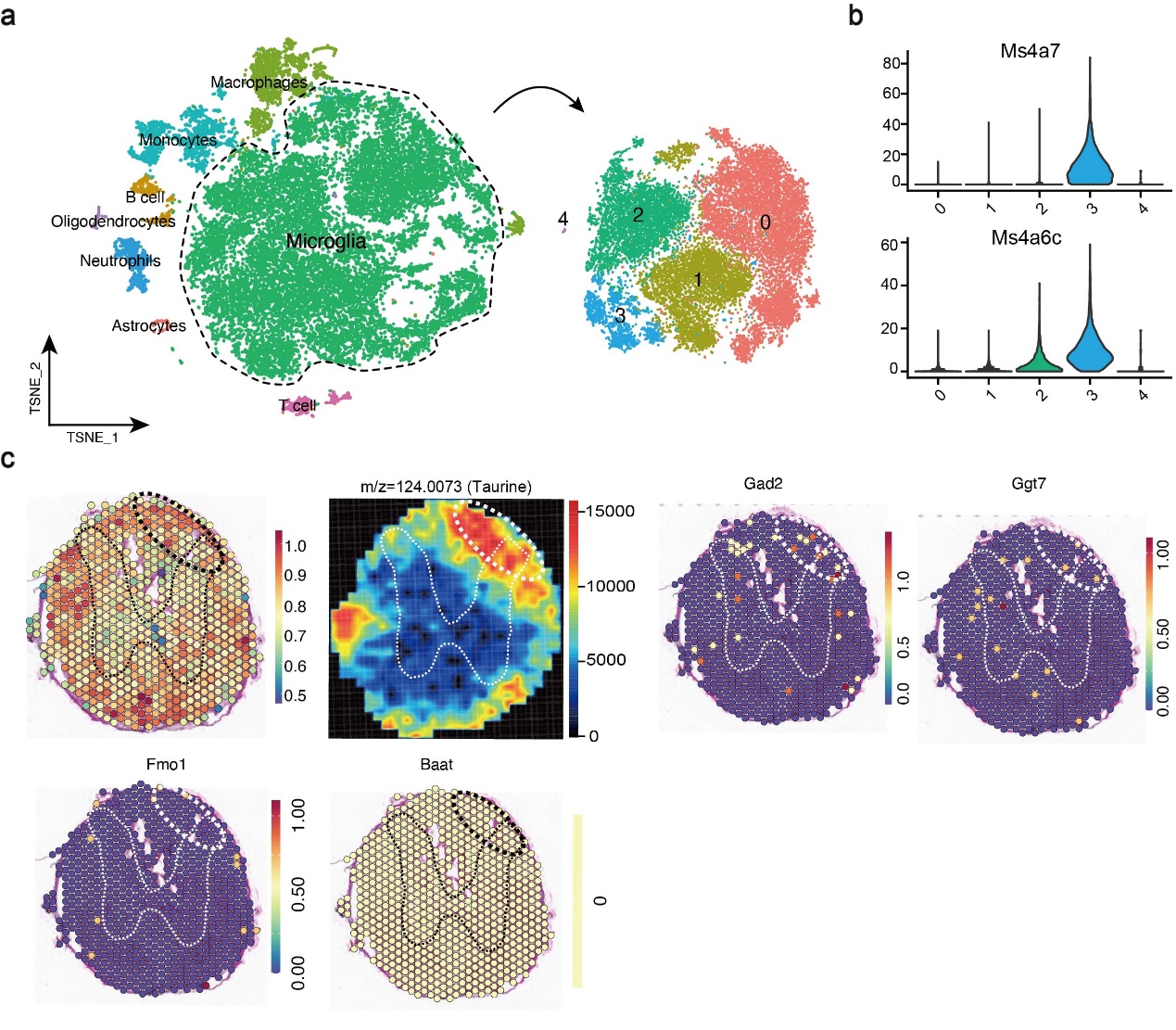


**Figure S6.** **Molecular and spatial characteristics of regeneration-promoting microglia.**

(**a** and **b**) Reanalysis showing the microglia subset 3 (MG3) is regeneration-promoting microglia.

(**c**) Comprehensive analysis including scRNA-seq, spital transcriptomics and spital metabolomics of Mic2 subset in SCI-03 sample. We circled the spinal cord gray matter for localization of spinal cord tissue. For both ST and SM maps, the color indicates the relative expression level of metabolites or genes, with red indicating higher level while blue indicating lower level of expression.


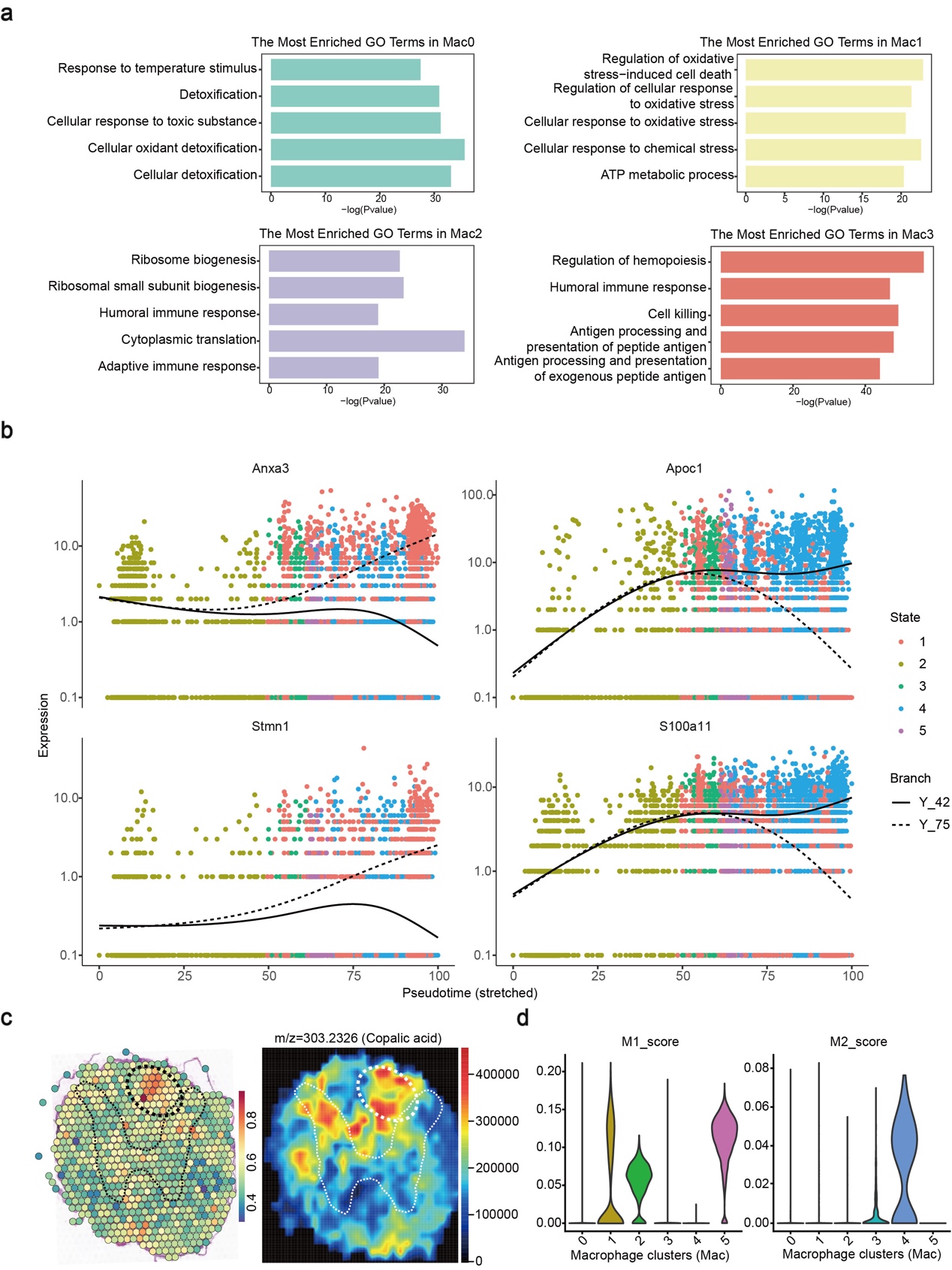


**Figure S7.** **Molecular and spatial characteristics of macrophage populations.**

(**a**) Top 5 enriched GO terms in Mac0-3, respectively.

(**b**) Scatter plots showing the expression changes of pro-inflammation genes (Apoc1, S100a11) and anti- inflammation genes (Anxa3, Stmn1) over time. The color indicates the different state and each point represents one cell.

(**c**) Comprehensive analysis including scRNA-seq, spital transcriptomics and spital metabolomics of Mac4 subset in SCI-01 sample. We circled the spinal cord gray matter for localization of spinal cord tissue. For both ST and SM maps, the color indicates the relative expression level of metabolites or genes, with red indicating higher level while blue indicating lower level of expression.

(**d**) Violin plots showing the M1 or M2 score in 6 macrophage subsets.


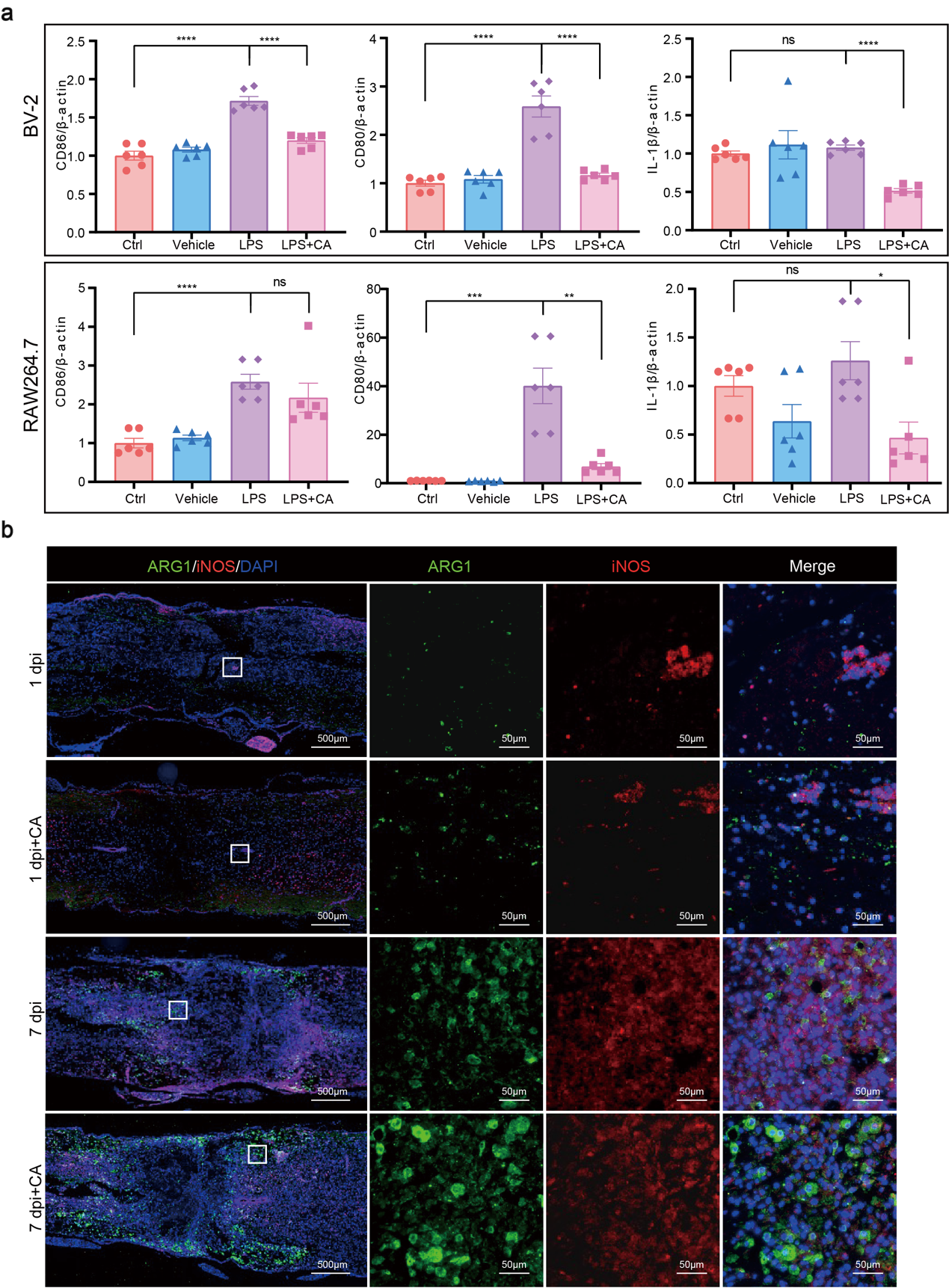


**Figure S8. Copalic acid inhibits inflammation after spinal cord injury.**

(a) The results of qRT-PCR showing the expression levels of pro-inflammation markers (CD86, CD80, IL-1β) in BV-2 and RAW264.7.

(b) Immunohistochemistry results reveal that ARG1 levels increase and iNOS levels decrease following treatment with copalic acid (CA) at both 1 day post-injury (dpi) and 7 dpi. Each group had six replicates. ^ns^ P>0.05, ^*^ P<0.05, ^**^ P<0.01, ^***^ P<0.001, ^****^ P<0.0001.


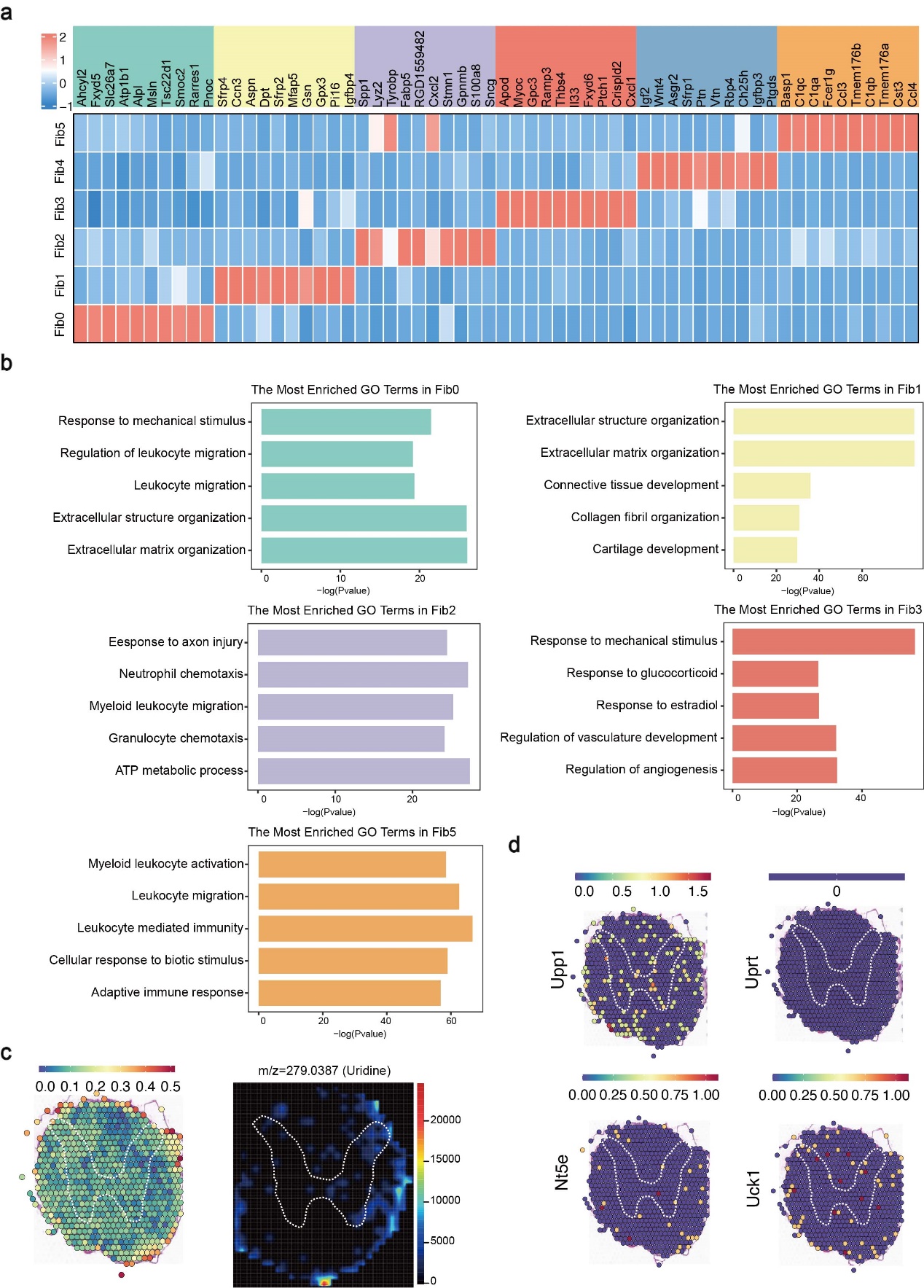


**Figure S9. Molecular and spatial characteristics of fibroblast populations.**

(**a**) Heatmap showing top 10 marker genes of 6 fibroblast subsets. The color indicates the relative expression level of marker genes, with red indicating higher level while blue indicating lower level of expression.

(**b**) Top 5 enriched GO terms in Fib0-Fib3, Fib5, respectively.

(**c** and **d**) Comprehensive analysis including scRNA-seq, spital transcriptomics and spital metabolomics of Fib4 subset in SCI-01 sample. We circled the spinal cord gray matter for localization of spinal cord tissue. For both ST and SM maps, the color indicates the relative expression level of metabolites or genes, with red indicating higher level while blue indicating lower level of expression.


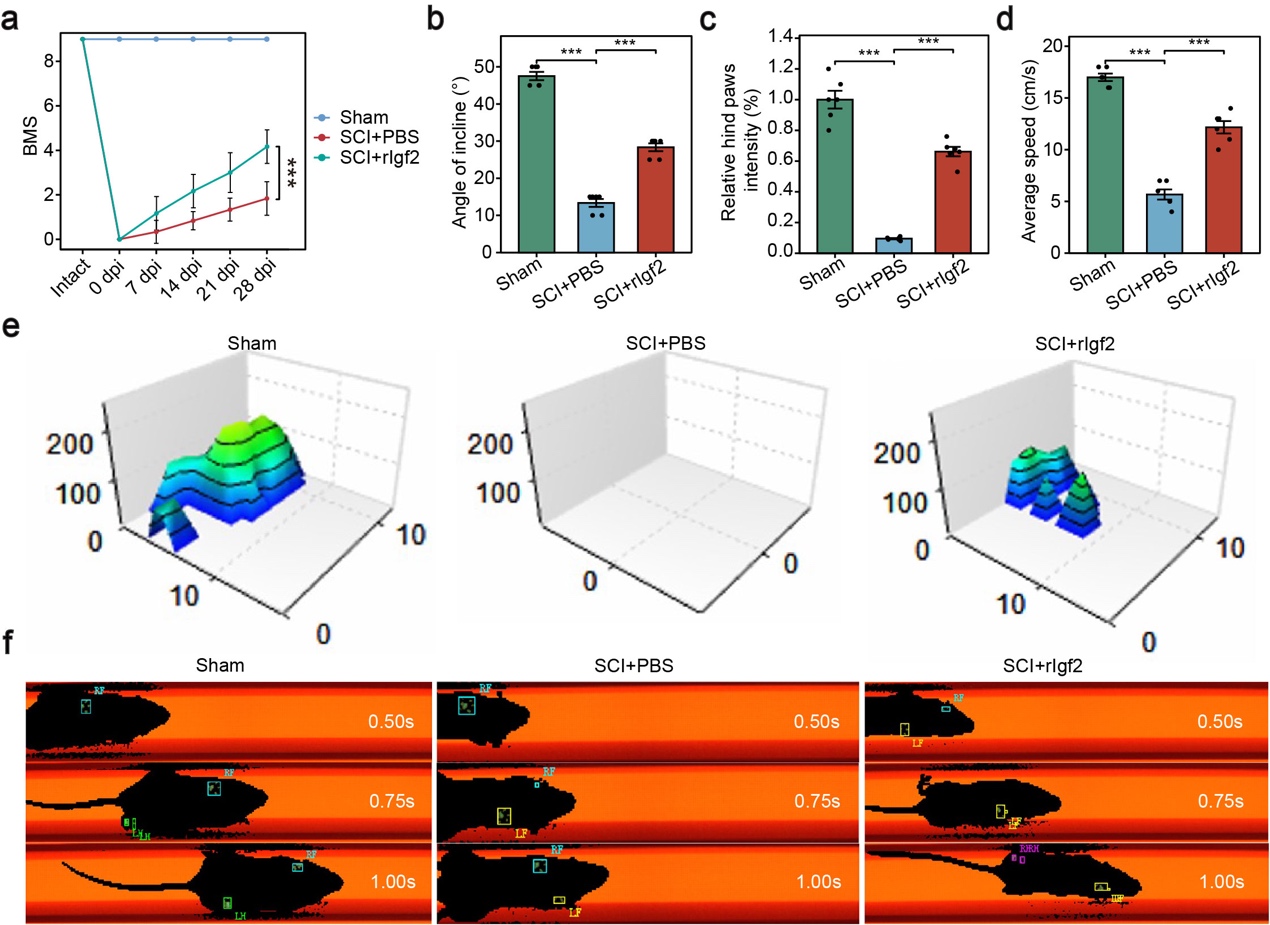


**Figure S10. Recombinant Igf2 (rIgf2) enhances functional recovery after SCI.**

(**a**) BMS scores over 28 days.

(**b**) Inclined plane test results.

(**c**) Hind paw pressure intensity.

(**d**) Average speed.

(**e-f**) Representative hind paw pressure intensity and stride patterns at 0.5s, 0.75s, and 1.00s intervals, showing improved coordination in rIgf2-treated mice. Each group had six replicates. ^***^ P<0.001.
